# First 4D-QSAR Study of Human Kynurenine 3 Monooxygenase (hKMO) Inhibitors: Integrating Chemical Space Networks and an Explainable Artificial Intelligence Platform for Neurodegenerative Disease Drug Discovery

**DOI:** 10.1021/acsomega.5c03404

**Published:** 2025-08-29

**Authors:** Sk. Abdul Amin, Joao Pedro Gallo Almeida Do Val, João Paulo Ataide Martins, Stefano Piotto

**Affiliations:** † Department of Pharmacy, 19028 Universita degli Studi di Salerno, Fisciano, Campania 84084, Italy; ‡ Departamento de Química, Universidade Federal de Minas Gerais, Belo Horizonte, MG 31270-901, Brazil

## Abstract

Human kynurenine 3-monooxygenase (hKMO) is a crucial enzyme in the kynurenine pathway (KP), which increases neurotoxicity by converting kynurenine into 3-hydroxykynurenine and quinolinic acid (QA)—both linked to oxidative stress and neuronal damage. KMO activity also reduces the neuroprotective metabolite kynurenic acid (KYNA), worsening disease progression. Inhibiting KMO counters these harmful effects since it restores KYNA levels, prevents toxic metabolite production, and reduces oxidative stress. This dual action makes KMO a vital therapeutic target in conditions such as neurodegenerative diseases, psychiatric disorders, acute pancreatitis, and immune dysregulation. In contemporary drug discovery, in silico design strategies offer significant advantages by revealing essential structural insights for lead optimization. The study is guided by three main objectives: (i) the development of a supervised machine learning (ML) model for a data set of hKMOis, (ii) chemical space networks (CSNs) analysis, and (iii) LQTA-QSAR (3D and 4D-QSAR) studies to generate interaction energy descriptors of Lennard-Jones (LJ) and Coulomb (C). To enhance accessibility, we present “phKMOi_v1.0,” a Streamlit-based web application accessible at https://phkmoiv1.streamlit.app/. This platform not only supports the prediction but also allows experts and nonexperts to interpret the key molecular features influencing KMO inhibitory activity through an interactive waterfall plot. These modeling analyses will assist medicinal chemists in designing more potent hKMOis in the future.

## Graphical abstract

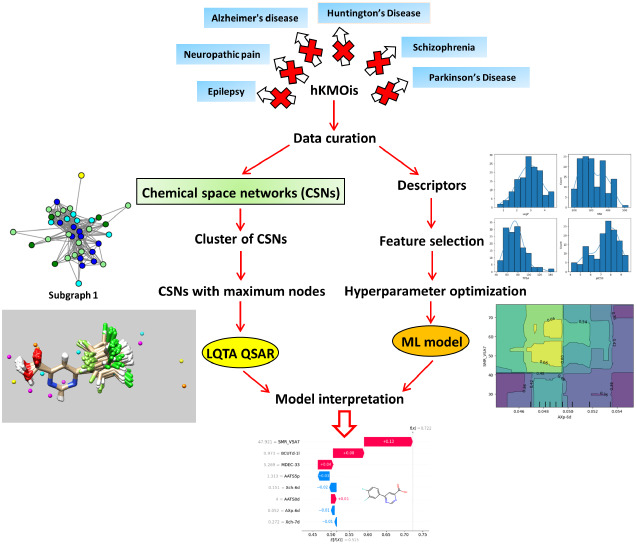

## 1 Introduction

Proteins and neurotransmitters, including melatonin and serotonin, are synthesized from the essential amino acid tryptophan.^1^ The kynurenine pathway (KP) metabolizes the majority of dietary tryptophan to produce nicotinamide adenine dinucleotide (NAD+), a critical energy cofactor. Two significant branches of the KP (Figure 1) are involved in synthesizing kynurenic acid (KYNA) and the neurotoxic quinolinic acid (QA).^2–4^

**Figure 1:**
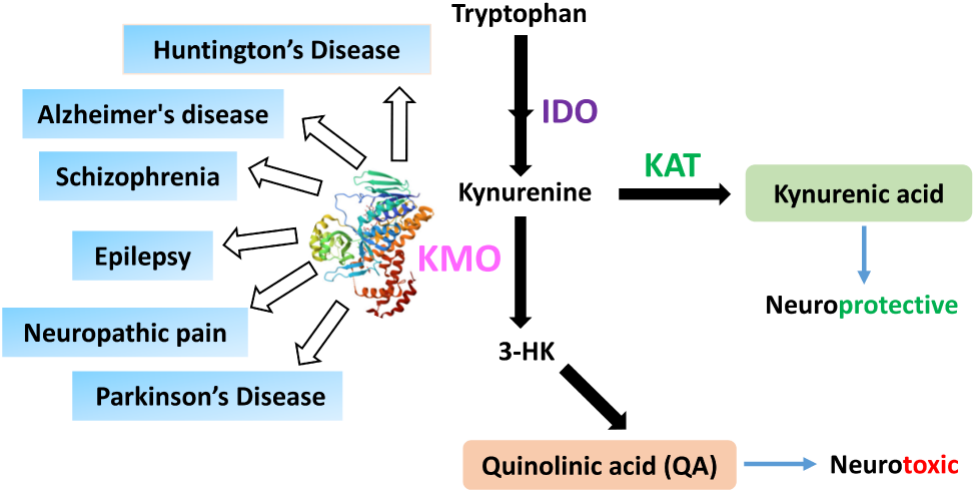
Graphical representation of the Kynurenine pathway. Kynurenine 3-monooxygenase (KMO) increases neurotoxicity by converting kynurenine into 3-hydroxykynurenine (3-HK) and quinolinic acid (QA)—both linked to oxidative stress and neuronal damage. KMO activity also reduces the neuroprotective kynurenic acid (KYNA). This dual action makes KMO a vital therapeutic target in several disease conditions (as depicted in cyan boxes).

Quinolinic acid (also known as QUIN), a neurotoxic NMDA (*N*-methyl-d-aspartate) receptor agonist, and kynurenic acid (KYNA), an NMDA receptor antagonist with neuroprotective properties, are both implicated in depression.^5,6^ The neurotoxicity of QA contributes to inflammation-induced neuronal and glial damage, accelerates neuronal death, reduces neuroplasticity, and induces depressive symptoms.^3^ Enzymes, namely kynurenine aminotransferase (KAT) and kynurenine 3-monooxygenase (KMO), utilize kynurenine (KYN) as a substrate in this pathway.^1–3,7–9^ By converting kynurenine into the neurotoxic metabolites 3-hydroxykynurenine (3-HK) and QA, KMO decreases the concentration of the neuroprotective metabolite KYNA while increasing levels of harmful metabolites and free radicals in the bloodstream.^9^ Numerous studies have associated KMO with brain and neurological diseases (Figure 1) such as Alzheimer’s disease,^10^ neuropathic pain,^11^ schizophrenia,^1,8,12^ Parkinson’s disease,^13^ and Huntington’s disease.^6,14,15^ Therefore, KMO represents a promising drug target for addressing neurodegenerative disorders.

The first crystal structure of human KMO (hKMO) was published in 2018;^16^ however, it is in an autoinhibited conformation (PDB: 5X68, https://www.rcsb.org/structure/5X68). Consequently, it is not the best choice to use for structure-based drug design (SBDD) purposes. While the X-ray crystal structure of hKMO remains unavailable, ligand-based drug design (LBDD) provides valuable insights into uncovering critical structural features for lead optimization. This study has three primary objectives, as shown in Figure 2.

**Figure 2:**
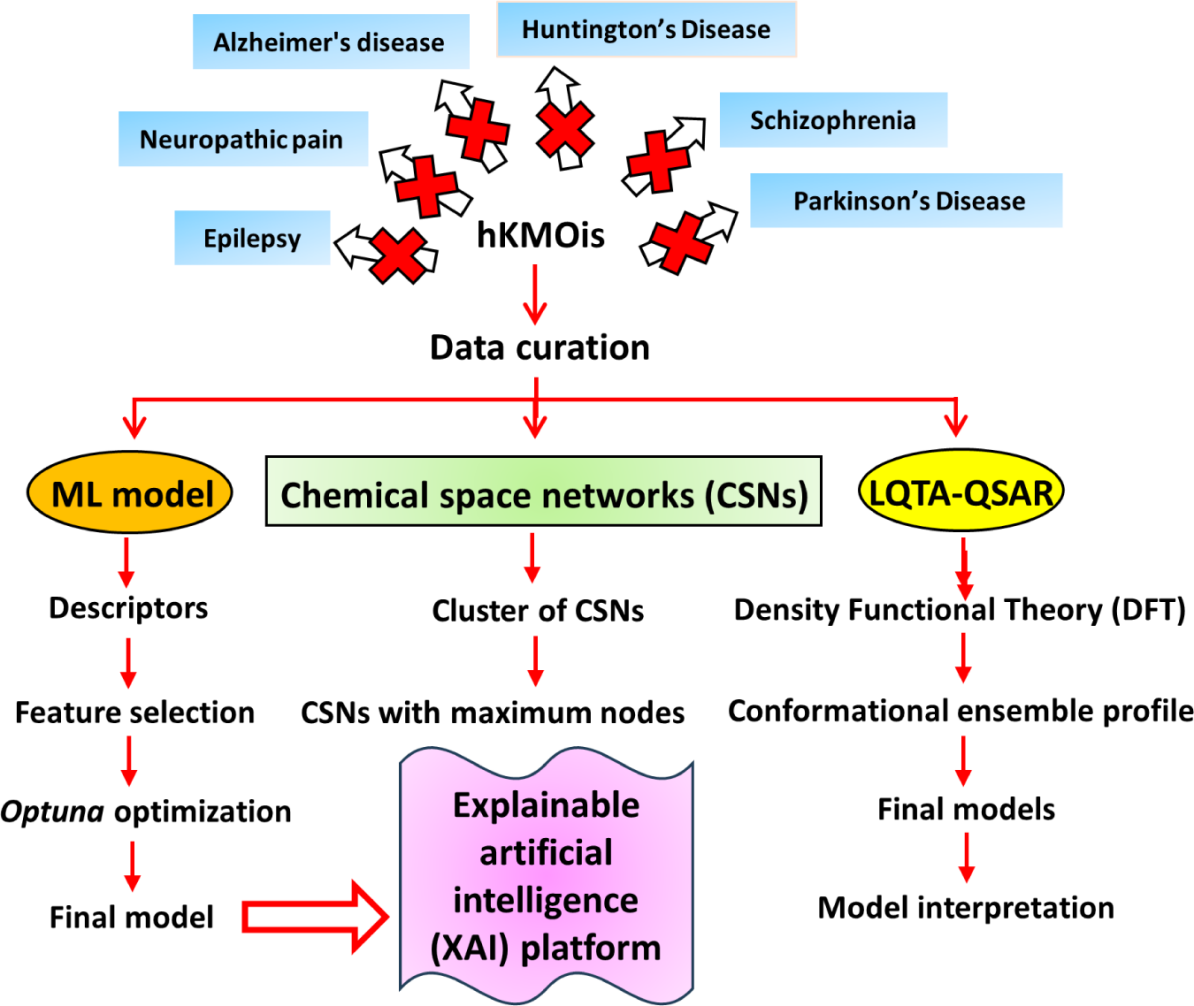
An overview of the study design. This study is an initiative to relate the structural requirements of hKMOis by supervised machine learning (ML) models, chemical space networks (CSNs), and Laboratório de Quimiometria Teórica e Aplicada (LQTA)-quantitative structure–activity relationship (QSAR) studies.

The main focus of the 2D-QSAR ML study is to provide valuable insights into the SARs of hKMOis using physicochemical descriptors, followed by the introduction of a Python-based web application named “**phKMOi_v1.0**” (available at https://phkmoiv1.streamlit.app/). This user-friendly platform not only enables the prediction of hKMO inhibitory activity but also allows both experts and nonexperts to interpret key molecular features through an interactive waterfall plot. In addition, the key advantage of 3D and 4D-QSAR models is their enhanced interpretability. These models can capture spatial features influenced by conformational flexibility, providing insights into their role in biological activities targeting hKMO. To the best of our knowledge, this is the first 4D-QSAR study addressing a neurological endpoint of hKMO inhibitory activity that integrates supervised ML models, an explainable artificial intelligence (XAI) platform, and CSNs.

## 2 Materials and Methods

### 2.1 Data Preparation

Biological activity data for hKMOis were sourced from the ChEMBL database,^17^ comprising small molecules with KMO inhibitory activities (IC_50_ values). After removing duplicates, entries lacking IC_50_ values, compounds with ranged values, and compounds without simplified molecular input line entry system (SMILES) annotations, the data set was refined to 137 hKMOis (Table S1).

### 2.2 Calculation of Pearson Correlation Coefficients

Each SMILES string was first parsed into an RDKit “Mol” object, followed by the calculation of eight physicochemical and topological descriptors using the “RDKit” (https://www.rdkit.org/) cheminformatics toolkit (version rdkit-pypi-2022.9.5).^18^ The physicochemical and topological descriptors were the octanol–water partition coefficient (*Log P*), molecular weight (*MW*), total number of ring systems in the molecule (*nRings*), number of aromatic rings (*nAR*), hydrogen bond acceptors (*HBAs*), hydrogen bond donors (*HBDs*), number of rotatable bonds (*nRBs*), and topological polar surface area (*TPSA*). The Pearson correlation coefficient was then computed to assess the linear relationships between these molecular descriptors and the biological activity (*pIC*_50_) of the hKMOis. Meanwhile, the Python scripts^19^ (File name: Suppplementary_NoteBook_KMO_Data set.ipynb) to calculate the Pearson correlation coefficients and other calculations are provided at https://github.com/Amincheminform/phKMOi_v1.

### 2.3 Machine Learning (ML) Study

#### 2.3.1 Data Set Division and Principal Component Analysis (PCA) Procedure

hKMOis with *pIC*_50_ < 7.6 were assigned as “*inactives*” (0), and compounds with *pIC*_50_ ≥ 7.6 were assigned as “*actives*” (1). After applying this cutoff, the data set contained 72 “*actives*” and 65 “*inactives*”. At first, the data set was randomly divided into training and test hKMOis (*N*_*Test*_ = 28). Then, circular fingerprints, specifically extended connectivity fingerprints with a radius of 2 and 2048 bits (*EFCP_4*), were computed for all hKMOis, followed by similarity analysis. Principal component analysis (PCA)^20^ was applied to reduce the high-dimensional similarity matrix to two dimensions. Subsequently, *k*-means clustering analysis (*k-*MCA)^21^ was conducted to identify chemical space groupings (*k* = 5, random seed = 42 to ensure reproducibility). Finally, a 2D PCA plot was generated to visualize these clusters and assess the distribution of training and test set compounds.

#### 2.3.2 Calculation of Features

Descriptors were calculated by using “Fiore_v1.0” platform, in particular the *Fiore_FC* (Feature Calculation) tool (https://github.com/Amincheminfom/Fiore_v1.0). This *Fiore_FC* tool used a molecular descriptor calculator named “Mordred”^22^ to calculate independent parameters (https://github.com/mordred-descriptor/mordred).

#### 2.3.3 Data Pretreatment and Feature Selection

The descriptors with non-numeric properties, missing values, and quasi-constant values were eliminated from the study as described earlier.^23^ Feature selection is a vital step in machine learning (ML) that enhances model performance. Initially, columns containing non-numeric values and constant columns are removed to eliminate noise and reduce dimensionality.^24^ To further refine the data set, measures such as information gain or mutual information are applied to identify the most relevant features for predicting the hKMO inhibitory activity. Only features with high mutual information scores are retained. Finally, features with importance scores greater than 0.120 are selected, optimizing accuracy while minimizing computational costs.

#### 2.3.4 Hyperparameter Tuning, Model Development, and Validation

Random Forest (RF), a supervised machine learning approach, is one of the most popular and versatile ML algorithms.^25^ Here, the hyperparameters of the RF classifier (RFC) for hKMOis were optimized by using *Optuna* (https://optuna.org/).^26^ The following hyperparameters were optimized within these ranges: (a) *n_estimators*: 10 to 100 (number of trees in the forest), (b) *max_depth*: 2 to 32 (maximum depth of each tree), (c) *min_samples_split*: 2 to 16 (minimum number of samples required to split an internal node), and (d) *min_samples_leaf*: 1 to 16 (minimum number of samples that a leaf node must have). The models were developed by “scikit-learn” (https://scikit-learn.org/stable/) package in Python language^27^ and were validated. Importantly, a 5-fold cross-validation strategy was employed during model training to ensure robustness and generalizability. This is a very common approach in the ML field. In this approach, the data set of hKMOis was partitioned into five equal subsets, and the RF classifier model was iteratively trained on four hKMOis subsets while being validated on the remaining hKMOis subset. This process was repeated five times, with each subset used exactly once as the validation set. The average performance across all folds was reported using the statistical metrics discussed earlier.^23^

#### 2.3.5 Applicability Domain Calculation and Analysis

Applicability domain (AD) analysis was performed using the leverage method^28^ to assess the reliability and generalizability of the developed RF model. First, the csv files of training and test data sets were taken, each containing selected descriptors. Then, training and test set descriptor matrices were concatenated to compute the leverage values for each hKMOi. The hKMOis with leverage values below this threshold were considered within the AD, while those exceeding the threshold were regarded as “outliers”. For better understanding, the leverage values for training and test compounds were visualized by using a scatter plot. The Python scripts to perform the applicability domain calculation and analysis are available at https://github.com/Amincheminfom/ML-study-of-non-hydroxamate-HDAC3i/blob/main/HDAC3_966_Optuna_En_v0.ipynb.

#### 2.3.6 Generation of Partial Dependence Plots

Partial dependence plots (PDPs) help to understand the influence of individual molecular descriptors on classification outcomes. In this study, PDPs were generated for important features by using *PartialDependenceDisplay* from “scikit-learn” (https://scikit-learn.org/stable/modules/generated/sklearn.inspection.PartialDependenceDisplay.html), with a grid resolution of 50 points per feature. Each partial dependence plot enables visualization of nonlinear relationships between the descriptor values and predicted activity probability. In addition, PDPs illustrate the marginal effect of a single feature on the predicted outcome by averaging over the values of all other features.^29^ The details are provided in Suppplementary_NoteBook_KMO_Data set (https://github.com/Amincheminform/phKMOi_v1).

#### 2.3.7 SHAP (SHapley Additive explanations) Plot

Further, SHAP (shapley additive explanations, https://shap.readthedocs.io/en/latest/) analysis^30^ was performed to gain more insights into the contribution of individual descriptors toward the classification outcomes of the trained supervised machine learning model for hKMOis. SHAP values were computed using the *TreeExplainer* class from the “shap” Python library (https://shap.readthedocs.io/en/latest/ generated/shap.TreeExplainer.html). A SHAP summary plot (also known as a beeswarm plot) was generated to visualize the global importance and distribution of feature effects across all training set hKMOis. This visualization helps us to find the most consistent descriptors that influence the decisions of the RFC model toward either class. Our Python scripts (File name: Suppplementary_NoteBook_KMO_Data set) to generate the beeswarm plot are available at https://github.com/Amincheminform/phKMOi_v1.

### 2.4 Chemical Space Networks

The relationships between the investigated hKMOis were explored through network analysis using “NetworkX” (https://networkx.org/)^31^ and “RDKit” (https://www.rdkit.org/)^18^ packages. Clusters were formed by using the greedy modularity community detection algorithm in “NetworkX”. In the CSNs,^32^ each node represents an hKMOi, while edges between nodes indicate structural similarity calculated using RDKit topological fingerprints (https://www.rdkit.org/ docs/GettingStartedInPython.html). The similarity between hKMOis was measured using the Tanimoto coefficient (*Tc*).^33^

### 2.5 LQTA-QSAR (3D and 4D-QSAR) Studies

A homogeneous subset of hKMOis was considered for LQTA-QSAR studies. The selected molecules were placed in a table with their respective SMILES and the negative logarithm of hKMO inhibitory activity (*pIC*_50_) for the first data treatment. Then, an *in-house* program (previously developed with the Python language^19^ (https://www.python.org/), the Open Babel package,^34^ and the program XTB^35^ (https://github.com/grimme-lab/xtb)) was used to convert the SMILES into 3D geometries, followed by semiempirical optimizations. After the optimization using the DFTB method (https://dftb.org/index.html), these molecules were optimized using *Ab Initio* methods, first with Hartree–Fock using the 6–31G** basis set and then with density functional theory (DFT) using the functional B3LYP and the cc-pVTZ basis set. These optimizations were carried out using a custom Python program built using the Psi4 library.^36^ Subsequent to the optimization phase, the LQTA-QSAR package^37^ was used. The 3D descriptors were calculated by using the optimized geometries obtained from DFT calculations aligned to the most active compound (K001). Likewise, the 4D descriptors were calculated from the conformational ensemble profile obtained from molecular dynamics (MD) simulations carried out according to the LQTA-QSAR methodology.^37^ With both sets of 3D and 4D descriptors, the *QSARModeling* package^38^ was used to build 3D and 4D QSAR models, respectively. Finally, the detailed descriptions and interpretations^39–41^ of the QSAR models were performed to enhance the understanding of the SARs of hKMOis.

## 3 Results and Discussion

### 3.1 Data Set

The investigated hKMOis were obtained from the ChEMBL database, followed by a refinement process (as described in Section 2.1), resulting in a final data set (Table S1). From the heatmap in Figure 3A, negative correlations are observed between molecular weight (*MW*) and *pIC*_50_, number of hydrogen bond acceptors (*HBAs*) and *pIC*_50_, number of hydrogen bond donors (*HBDs*) and *pIC*_50_, number of rotatable bonds (*nRBs*) and *pIC*_50_, topological polar surface area (*TPSA*) and *pIC*_50_ (Figure 3A). In contrast, positive correlations are observed between lipophilicity (*Log P*) and *pIC*_50_, number of aromatic rings (*nARs*) and *pIC*_50_, and number of rings (*nRings*) and *pIC*_50_.

**Figure 3:**
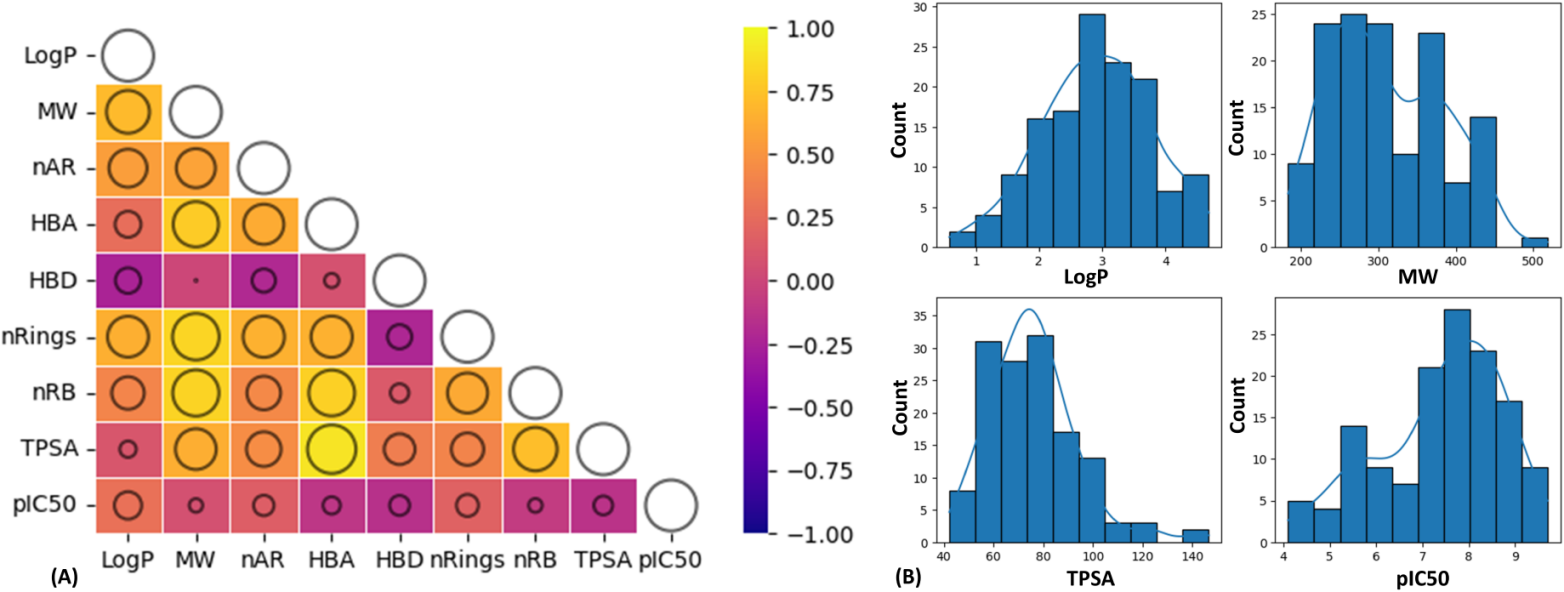
(A) The heatmap of Pearson correlation coefficients between pairs of features (data set: hKMOis). The bubble sizes correspond to the absolute values of the correlation coefficients, where larger bubbles represent stronger correlations. (B) Bin plots of *Log P*, *MW*, *TPSA,* and *pIC*_50_ features.

The Pearson correlation coefficient (*r*) value of *Log P* and *pIC*_50_ is 0.29, indicating that molecules with lipophilic characteristics are moderate to good hKMOis. The mean *Log P* value across all data set molecules is 2.901 (Figure 3B), suggesting most molecules are moderately lipophilic. The highest and lowest *Log P* values are found to be 4.687 and 0.579, respectively, indicating the most lipophilic and most hydrophilic hKMOis, respectively, in the data set. The average *MW* (310.145) of molecules indicates that most of the molecules are small to medium-sized. The mean *TPSA* value across all hKMOis is 75.920. On average, molecules have two aromatic rings and two or three rings. Notably, hKMOis have around four rotatable bonds, indicating moderate to lower flexibility.

### 3.2 Machine Learning (ML) Study

hKMOis with *pIC*_50_ < 7.6 were assigned as “*inactives*” (0), and compounds with *pIC*_50_ ≥ 7.6 were assigned as “*actives*” (1). The PCA scatter plot is depicted in Figure S1A. hKMOis belonging to the training and test sets were marked by using red and blue markers, respectively, to highlight their distribution across chemical space. This visualization confirmed that the training and test compounds were well distributed across the different chemical clusters, indicating that the random splitting preserved the overall diversity of the data set.

#### 3.2.1 Descriptors

Descriptors were calculated by using “Fiore_v1.0” platform, in particular the *Fiore_FC* (Feature Calculation) tool (https://github.com/Amincheminfom/Fiore_v1.0). Then, the descriptors with non-numeric properties, missing values, and quasi-constant values were eliminated from the study, as described earlier.^23^ From an initial set of 1614 descriptors, 37 descriptors with importance scores exceeding 0.120 were chosen. Finally, 8 descriptors (*MDEC-33*, *AXp-6d*, *BCUTd-1l*, *SMR_VSA7*, *Xch-7d*, *Xch-6d*, *AATS0d*, and *AATS5p*) were selected for ML studies based on their feature importance values.

#### 3.2.2 Results of the Random Forest (RF) Model

The hyperparameters of the RF model^25^ were optimized using *Optuna*.^26^ The model tuning led to the selection of the best hyperparameters (*n_estimators*: 18, *max_depth*: 7, *min_samples_split*: 4, *min_samples_leaf*: 1), which optimize the performance of the RF model (Figure 4). The *n_estimators* parameter was found to be 18, which means that 18 trees were determined to be optimal. A larger number of trees usually improves the performance and stability of the model but also increases the computational cost (Figure 4A).

**Figure 4:**
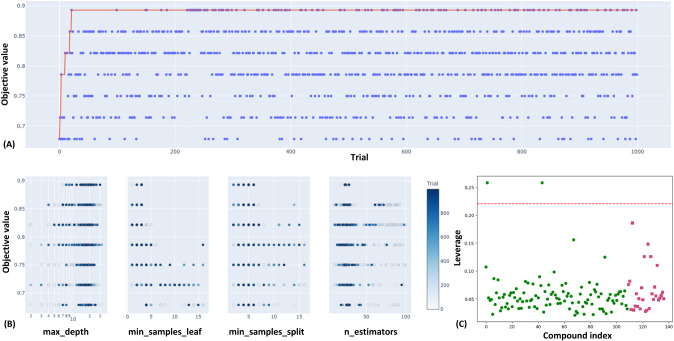
(A) Optimization history plot of the hyperparameter optimization process for finding the optimal values (1000 runs). (B) Slice plot of specific hyperparameters, namely *max_depth*, *min_samples_leaf*, *min_samples_split*, *n_estimators*. (C) Plot of the applicability domain (AD) for hKMOis.

A depth of seven indicates that each tree can grow to seven levels (Figure 4B). Altogether, it can be stipulated that the selected hyperparameters optimize the predictability of the RF model while avoiding overfitting. This model correctly classified 89.29% of the instances (precision of 87.50%, recall of 93.33%, and F1 score of 90.32%, as depicted in Table 1) in the validation data set, indicating its ability to capture generalizable patterns.

**Table 1:** Model Performance for the Data Set of hKMOis^a^

Set	Accuracy	Precision	Recall	F1 Score
**Training**	0.9358	0.9310	0.9474	0.9391
**Test**	0.8929	0.8750	0.9333	0.9032

a *n_estimators*: 18, *max_depth*: 7, *min_samples_split*: 4, *min_samples_leaf*: 1.

Notably, the recall remains consistently high across cross-validation, training, and test sets, which is particularly important when the objective is to accurately identify “*actives*” hKMOis for early-stage drug discovery. The receiver operating characteristic (ROC) plot of the test set (area under the curve, AUC: 0.9077) is given in Figure S1B. In addition, three ensembles of train and test sets have also been considered to build new RF models. The results are found to be comparable with the original model, suggesting the robustness of the developed RF model.

The feature importance plot of the selected descriptors is shown in Figure S1C. *MDEC-33* is a molecular distance edge (MDE) descriptor related to the MDE between all tertiary carbons. The descriptor, *AXp-6d*, belongs to the atom-type electrotopological state (E-State) index family. It represents atomic contributions to the molecular properties and topology of hKMOis. A *BCUT* (Burden-CAS-University of Texas) descriptor, *BCUTd-1l*, evaluates molecular properties such as size, shape, and electronic distribution, focusing on low eigenvalues. *SMR_VSA7* is a surface area descriptor weighted by molar refractivity (SMR). Moreover, *Xch-7d* and *Xch-6d* are molecular connectivity chi indices descriptors. These are used to capture aspects of the molecular topology and branching. A descriptor from the average atom-type electrotopological state (AATS) series, *AATS0d*, represents an average of electrotopological state values for atoms in the molecule. *AATS 5p* (Average Broto-Moreau autocorrelation—lag 5/weighted by polarizabilities) is similar to *AATS0d* but considers the average atom-type values with specific weighting (e.g., polarizabilities).

LBDD has seen significant advances in recent years, particularly with the integration of perturbation-theory machine learning (PTML) modeling, enabling simultaneous prediction of biological activity against multiple targets. Notably, PTML has been successfully applied to the design of novel chemotypes, including multitarget inhibitors for GSK3B/HDAC1/HDAC6 (Alzheimer’s disease)^42^ and SERT/NET (mood disorders).^43^ While our present statistically validated ML model focuses on single-target prediction of hKMOis, it complements the PTML paradigm by offering a transparent, interpretable, and easily accessible platform that supports the early-stage consideration of the scaffolds targeting hKMO.

#### 3.2.3 Applicability Domain (AD) Analysis

Here, the leverage approach (number of descriptors = 8, number of training sets = 109) was used to identify X-outliers within the training set and to detect molecules outside the AD when applied to the test set.^28^ The results indicate that all of the test set hKMOis are within the range of AD [compounds within the applicability domain (test) = 28]. Hence, no outlier [compounds outside the applicability domain (test) = 0] is found in the test set compounds (threshold value of 0.220). Notably, only two hKMOis (Figure 4C) in the training set are found to be outliers: K067, leverage value: 0.258, and K068, leverage value: 0.258.

#### 3.2.4 Partial Dependence Plots

Partial dependence plots (PDPs) for the top four molecular descriptors used in the RF classifier are depicted in Figure 5. Each plot in Figure 5 illustrates the marginal effect of single features such as (A) *AXp-6d*, (B) *BCUTd-1l*, (C) *AATS5p*, and (D) *SMR_VSA7* on the predicted probability of an hKMOi being classified as “*actives*”, while averaging out the effects of other descriptors.

**Figure 5:**
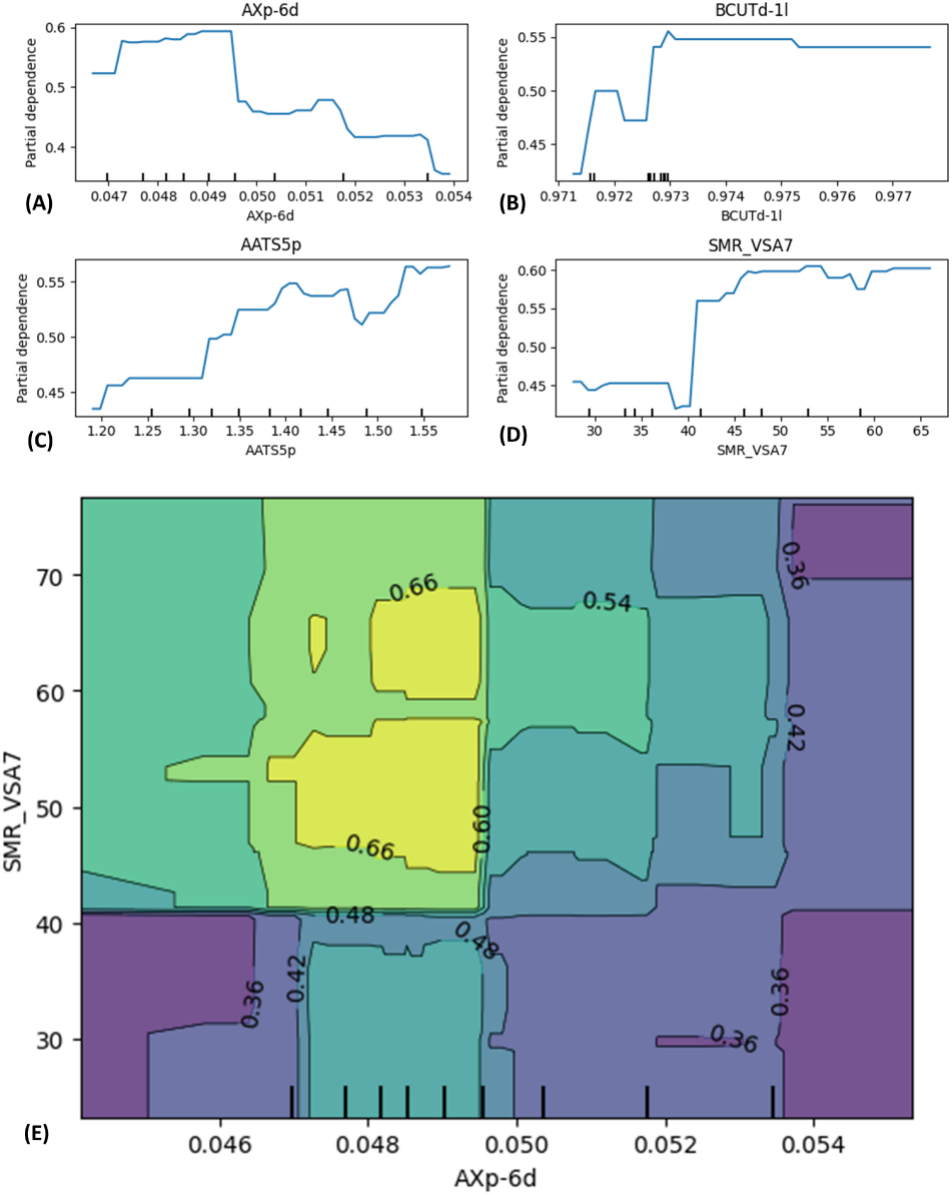
Partial dependence plot (PDP) of (A) *AXp-6d*, (B) *BCUTd-1l*, (C) *AATS5p*, and (D) *SMR_VSA7*. The *x*-axis represents the range of descriptor values observed in the training data. (E) Two-variable PDP of chemical descriptors *AXp-6d* vs *SMR_VSA7*.

*AXp-6d* quantifies molecular topological complexity along 6-atom paths. The narrow range (from 0.04 to 0.06) of *AXp-6d* suggests subtle but important variations in the molecular topology (Figure 5A) associated with the hKMO inhibitory activity. A BCUT descriptor, *BCUTd-1l*, reflects the atomic partial charge and topological character. The range from 0.91 to 0.99 indicates discriminative electrostatic diversity across the data set (Figure 5B). Next, the range from 1.12 to 1.62 of the *AATS 5p* captures midrange molecular flexibility and polarizability effects (Figure 5C). Furthermore, the sum of van der Waals surface areas for atoms falls within a specific molar refractivity range (23.22 to 76.66 in Figure 5D), reflecting contributions of size and lipophilicity. Understanding the ranges of these descriptors is important to explore the impact of molecular features on the behavior of the model. Additionally, a two variable PDP of selected chemical descriptor *AXp-6d* vs *SMR_VSA7* is also plotted (Figure 5E). The contour plot color-codes regions are based on numerical values, as indicated by the contour levels. It seems highest in the yellow-green region and lower in the darker blue and purple areas. The colors range from darker to lighter shades, where lighter regions seem to correspond to higher values. In the plot of Figure 5E, lighter regions correspond to higher predicted probabilities, indicating a higher likelihood of the compound being classified as “*actives*”. *SMR_VSA7* represents the van der Waals surface area contributions from atoms with moderate molar refractivity values. Values above 41 for these features likely indicate compounds with more surface area and potentially enhanced lipophilic interactions. There seems to be a significant region around *AXp-6d* of 0.047 to 0.050 and *SMR_VSA7* of above 41, where the performance metric reaches its high (Figure 5E) prediction scores from the RF model. Thus, the model appears to associate moderate topological complexity (*AXp-6d* ≈ 0.047 to 0.050) and increased lipophilic surface area (*SMR_VSA7* > 41) with a higher probability of compound activity.

#### 3.2.5 SHAP (Shapley Additive Explanations) Plot

The SHAP (shapley additive explanations, https://shap.readthedocs.io/en/latest/) summary plot of SHAP values for each descriptor of the training set is given in Figure 6A. The color shift from blue to red indicates that the descriptor ranges from low to high values. Color encodes the feature value (red = high, blue = low) of hKMOis. This helps to understand the magnitude of a descriptor and the influences of the feature in the prediction of the ML model. For instance, *Xch-6d*, high values (red) are typically associated with positive SHAP values, meaning that higher topological complexity promotes KMO inhibitory activity.

**Figure 6:**
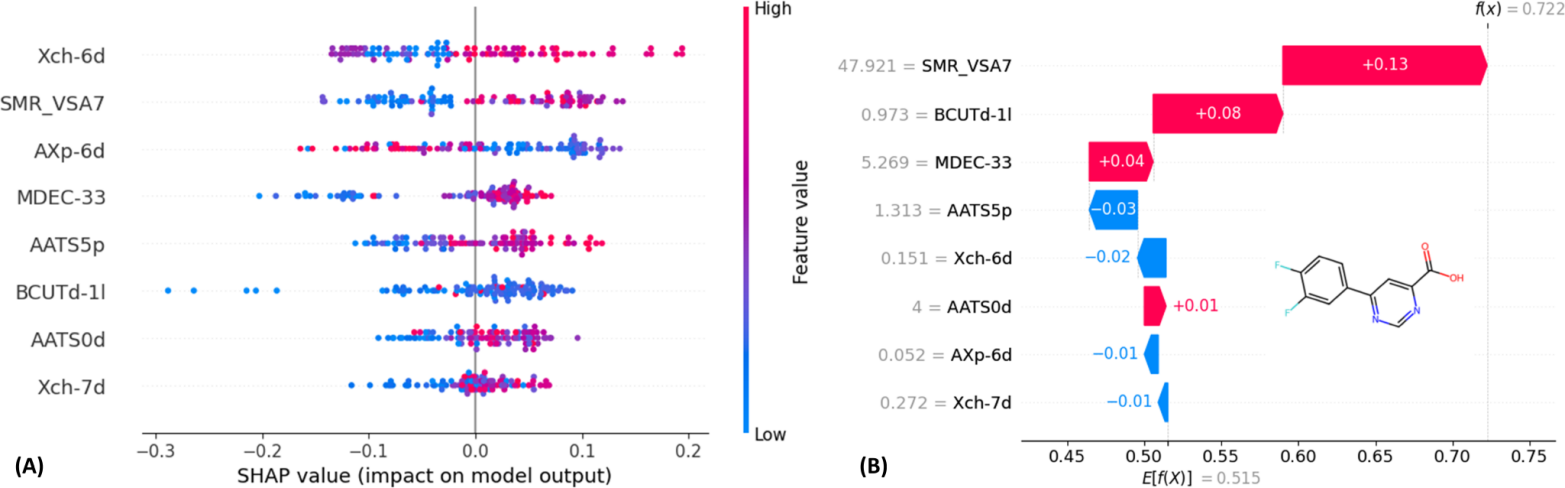
(A) Summary plot of SHAP values for each descriptor of the training set. (B) Waterfall plots of the most active molecule from this investigated data set.

Conversely, for *BCUTd-1l*, low values (blue) may correspond to negative SHAP values, indicating that less favorable electrostatic properties shift the prediction toward inactivity. *SMR_VSA7* shows a wider spread of both positive and negative SHAP values, suggesting that its influence is dependent on its specific value and interaction with other features. Importantly, the mean absolute SHAP value for each descriptor (horizontal axis ranking) quantifies the contribution of the descriptors to the model on average across all predictions. Descriptors with higher mean absolute SHAP values are therefore more consistently impactful in determining activity.

Additionally, the SHAP waterfall plot (Figure 6B) illustrates the contributions of individual features in the final model prediction for a single compound (for example, the most active molecule from this investigated data set). The *x*-axis of Figure 6B reports *E*[*f*(*x*)], which stands for the expectation function over all sums of SHAP values calculated on the training set hKMOis. Features contributing positively (bars extending to the right) increase the probability of classifying the hKMOi as “*active*”, while features contributing negatively (bars extending to the left) reduce that probability, pushing the prediction toward “*inactive*”. From the plot in Figure 6B, it can be observed that *SMR_VSA7* provides the largest positive contribution, significantly increasing the prediction toward the “*active*” class for the investigated molecule. The next important descriptors, BCUTd-1l and *MDEC-33*, exhibit good contributions, while the *AATS 5p*, *Xch-6d* have poor contributions for the investigated molecules.

### 3.3 Explainable Artificial Intelligence (XAI) Platform to Unveil the Molecular Rationale Behind KMO Inhibition

The primary format is a Streamlit (https://streamlit.io)-based web application accessible at https://phkmoiv1.streamlit.app/. Users can input an SMILES string of their query molecule to predict its KMO inhibitory activity. This platform predicts the outcomes for external data (query molecules) using our pretrained ML model and provides a detailed explanation of the predictions using SHAP analysis (Figure 7). It visualizes both the SHAP explanation and the structure of (a) the query molecule, (b) the most similar molecule from the data set with respect to the query molecule, and (c) the most active molecule from the data set.

**Figure 7:**
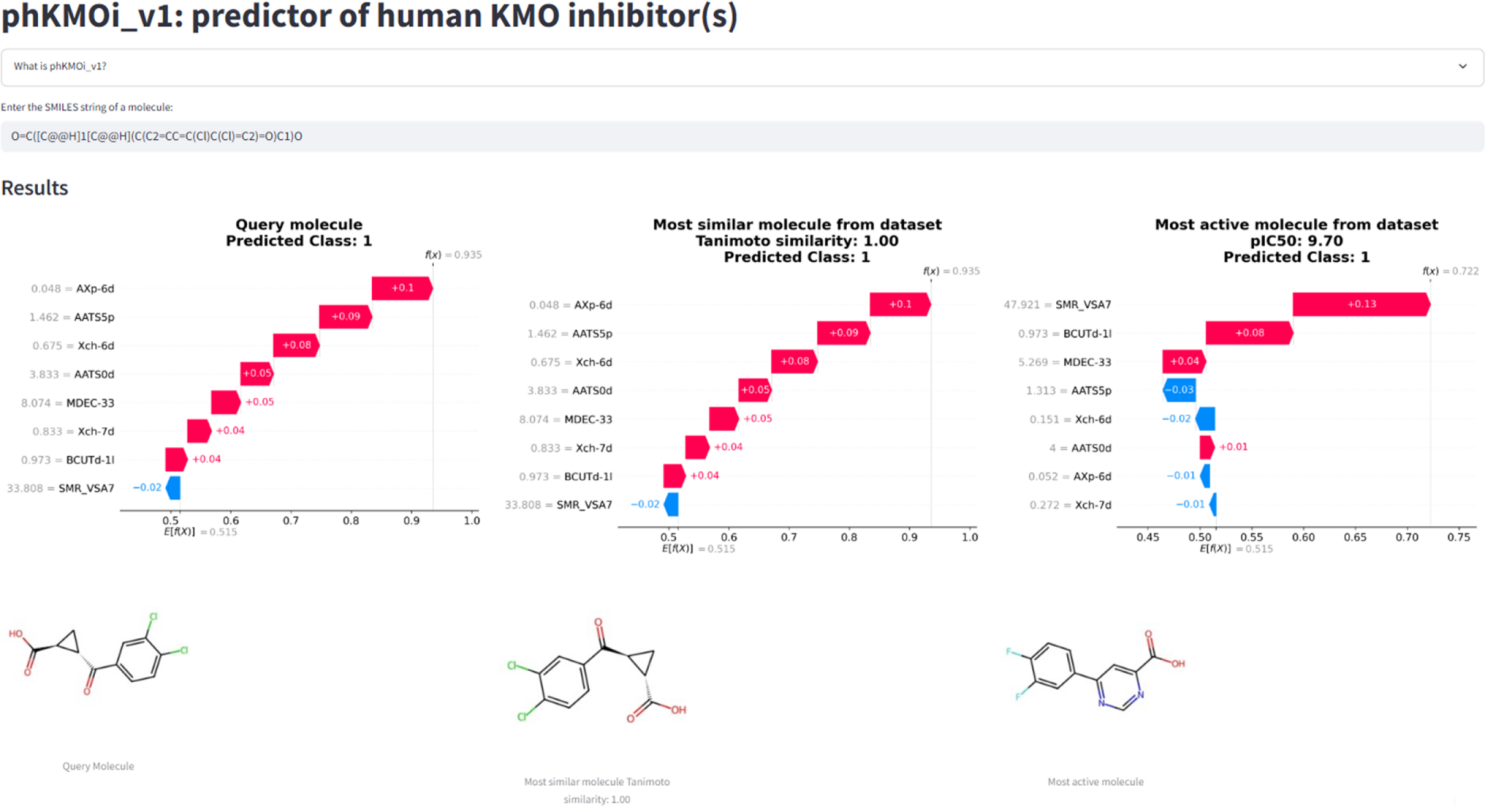
Interface of the “**phKMOi_v1.0**” tool. The visualization of 2D structures and the SHAP waterfall plots of the query molecule, the most similar molecule from the data set with respect to the query molecule, and the most active molecule from the data set.

In Figure 7, an example of the prediction of a known hKMOi via the “**phKMOi_v1.0**” tool is depicted. Since the query molecule was already present in the data set, the most similar molecule from the data set with respect to the query molecule is the same one (similarity = 1). This tool/XAI platform perfectly predicted the known hKMOi as “*active*” (Class: 1). Hence, this tool predicted the hKMOi with an external set accuracy of 0.8929. Another example of a prediction with an unknown query molecule is given in Figure S2. Importantly, this user-friendly XAI platform serves two key purposes:

(a) *Easy accessibility and knowledge spreading*: The visualization of 2D structures and the SHAP waterfall plots of the query molecules along with the known “*active*” hKMOi together offer an interpretable view of the features contribution to the KMO inhibitory prediction. This understanding might enhance the decision-making process.
(b) *Hypothesis generation for future research*: This platform also encourages rational design of new chemical scaffolds from the descriptor interpretations and SAR trends that potentially accelerate the drug discovery efforts for hKMO inhibition, especially in the absence of large-scale screening data.

### 3.4 Chemical Space Network (CSN) Analysis

We applied CSN analysis^32^ to unveil the networks with nodes (representing hKMOis) and edges (representing structural similarity). Here, structural similarity was calculated by using the Tanimoto coefficient (*Tc*) with RDKit topological fingerprints, and edges were drawn between nodes (hKMOis) if *Tc* ≥ 0.68. Meanwhile, this analysis revealed 14 subgraphs (or clusters), where 4 subgraphs (1, 2, 4, and 12) contain more than 10 hKMOis (Figure 8). The remaining clusters possess two or fewer hKMOis (Figure S3). All of the clusters together can be visualized in Figure S4.

**Figure 8:**
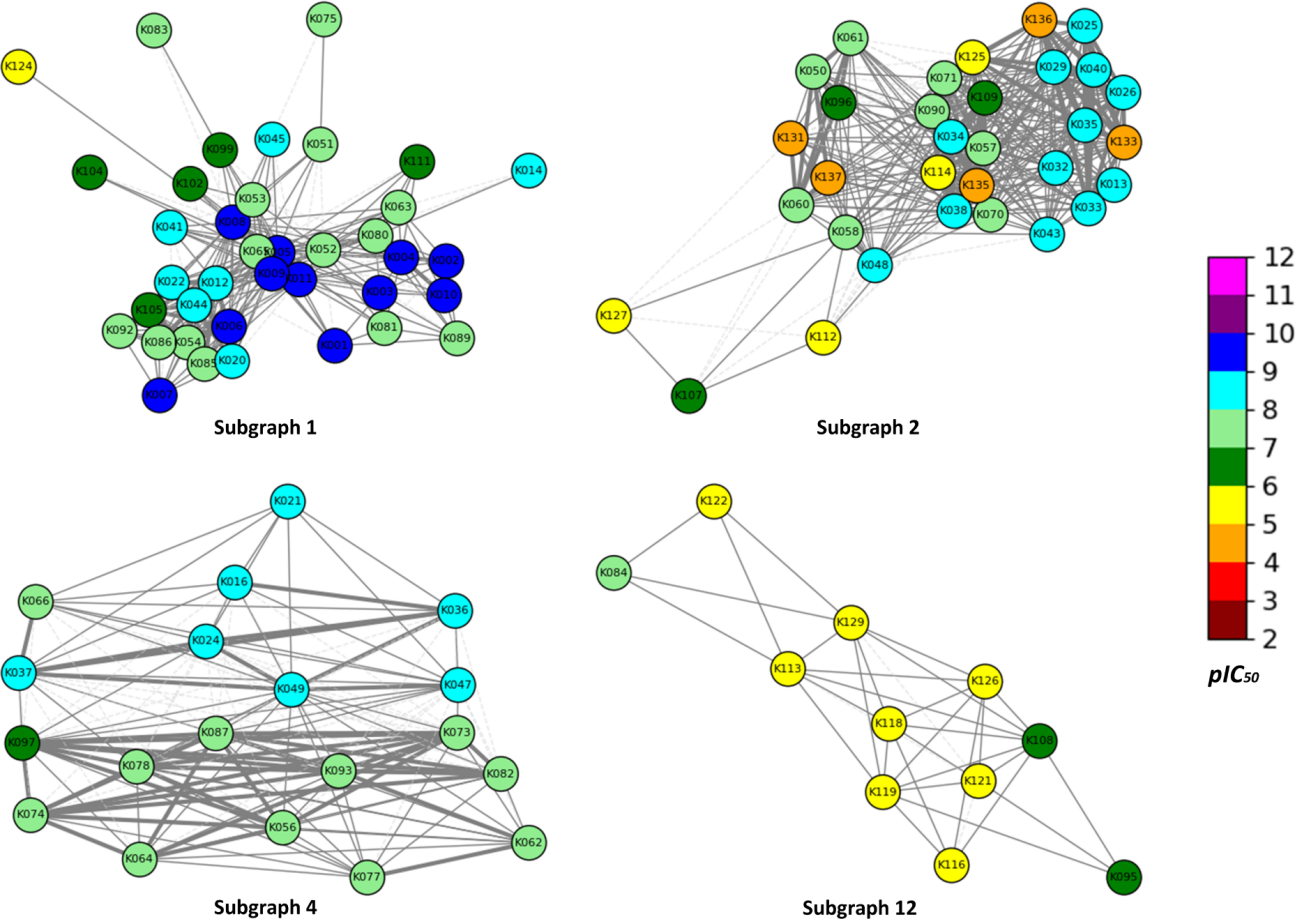
A spring layout CSN component of 4 clusters (denoted as subgraphs 1, 2, 4, and 12) of hKMOis. Molecule ID is also highlighted for better understanding. The color of the nodes represents the *pIC*_50_ values (as per the color map) of the hKMOis, and the line style in the network graph is dependent on the *Tc*-based similarity value. Thick lines for excellent similarity (*Tc* ≥ 0.9); medium lines for high similarity (0.7 < *Tc* < 0.9); thin lines for moderate similarity (*Tc* ≤ 0.7).

A list of hKMOis (with their molecule IDs) belonging to subgraphs 1, 2, 4, and 12 is provided in Table S2. Moreover, Table S3 summarizes the structural similarities and differences among the hKMOis within subgraph 1, based on edge weights representing pairwise similarity (*Tc* ≥ 0.68). In subgraph 1 (Figure 8), 29 pairs of hKMOis possess a similarity of ≥ 0.9, indicating that 10.94% of the pairs are excellenty similar. Likewise, 21.47% (Table S4) and 29.71% (Table S5) of the pairs are excellenty similar (*Tc* ≥ 0.9) in subgraphs 2 and 4, respectively. Notably, in subgraph 12, 17.65% of the pairs exhibit high similarity with *Tc* ≥ 0.8 (Table S6). For further analysis, we focused on subgraph 1 (CSNs with maximum hKMOis).

### 3.5 LQTA-QSAR Studies

The final 3D-QSAR model is obtained based on five latent variables (*nLV*). The coefficient of determination is found to be 86.97%. It means that 86.97% of the variance in the training set is explained by this model (Table S7). The root mean square error of calibration (*RMSEC* = 0.399) measures the promising quality of the model. Lower values indicate better calibration.^40^ The prediction residual sum of squares (*PRESS*), indicating the sum of squared errors from cross-validation, is associated with *Q* ^2^ values of 0.812. Moreover, the value of *F* = 42.707 confirms that the model is statistically robust. Good external validation is also suggested by the *R* ^2^_*Pred*_ values.^41^ Figure 9 presents the Lennard-Jones (LJ) and Coulomb (C) descriptors selected in the chemical space around the most active (Figure 9A) and the poorly active molecule (Figure 9B).

**Figure 9:**
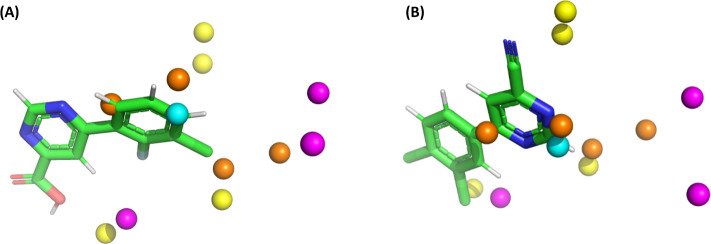
Most active K001 (A) and poorly active K124 (B) compounds with selected descriptors (as per the 3D-QSAR model) around them. Yellow: Lennard-Jones descriptor with a positive regression coefficient; magenta: Lennard-Jones descriptor with a negative regression coefficient; cyan: Coulomb descriptor with a positive coefficient; orange: Coulomb descriptor with a negative coefficient.

Molecular descriptors are obtained from the conformational ensemble profile (CEP) for the RI-4D-QSAR study using the Laboratório de Quimiometria Teórica e Aplicada (LQTA)-QSAR methodology.^37^ Notably, the 4D-QSAR model (*nLV* = 2) is good enough to explain 91.96% of the variance and generate the model with a low standard error of calibration (*SEC* = 0.335). Here, the *Q* ^2^ and root mean square error of cross-validation (*RMSE*_*CV*_) are found to be 0.866 and 0.433, respectively. The *F* value obtained here for this 4D-QSAR model is 154.371. The predictive residual sum of squares of cross-validation (*PRESS*_*CV*_) value of 5.636 (Table S7) suggests the quality of the model.

LJ and C descriptors selected around the CEP of the most active and the least active molecules from subgraph 1 are shown in Figure 10. The C descriptors with positive coefficients are found to contribute favorably to the model, suggesting the importance of halogens in enhancing the KMO inhibitory activity. Two LJ descriptors are located near the pyrimidine ring of K001 (Figure 10), implying that introduction of bulky groups with electrostatic properties may not be beneficial for the hKMO inhibitory activity.

**Figure 10:**
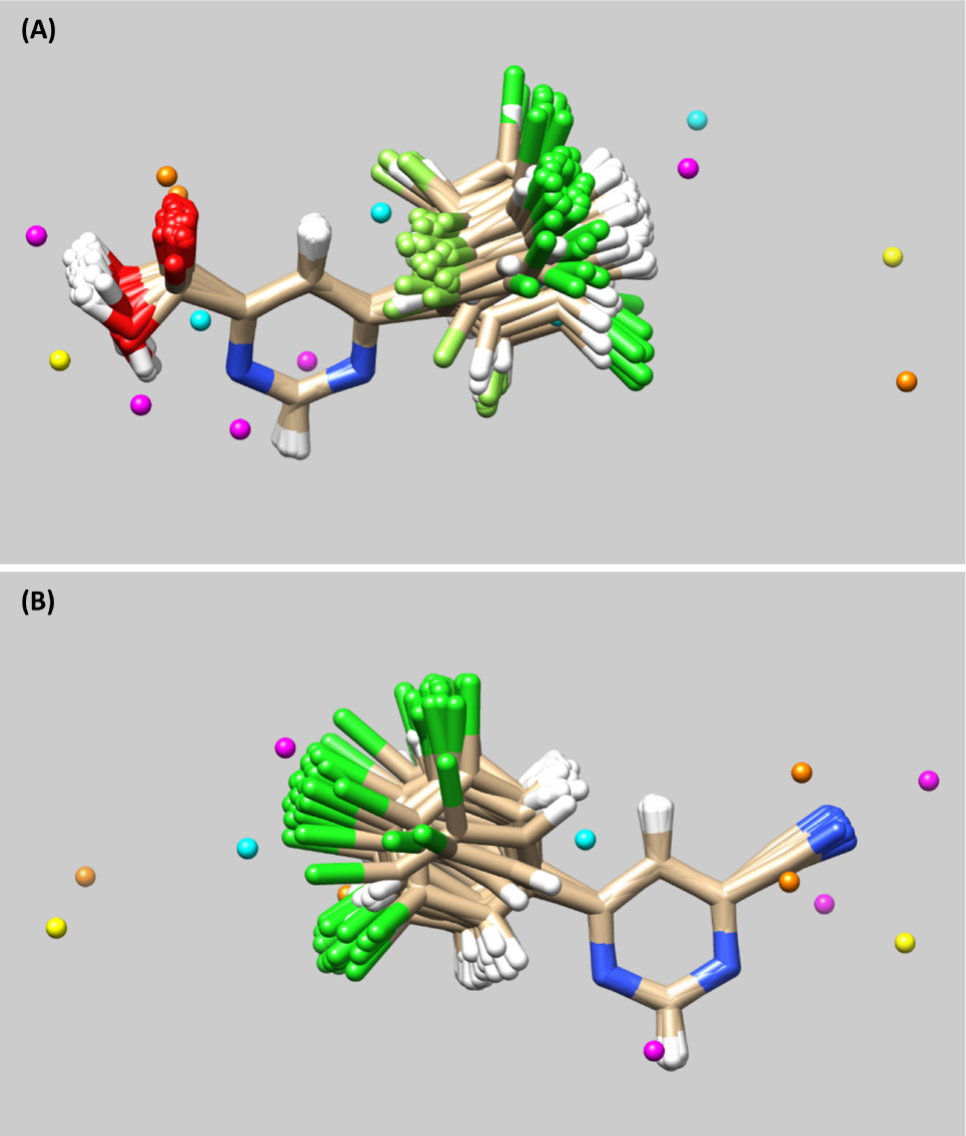
Compounds K001 (A) and K124 (B) with selected descriptors (as per the 4D-QSAR model) around the conformational ensemble profile (CEP) of each ligand. Yellow: Lennard-Jones descriptor with a positive regression coefficient; Magenta: Lennard-Jones descriptor with a negative regression coefficient; cyan: Coulomb descriptor with a positive coefficient; orange: Coulomb descriptor with a negative coefficient.

This also describes the structural information corresponding to the shape and conformational flexibility of the ligands, which indicates that the bulky group at that position is unfavorable for biological activity (Figure 11). These observations agree with the SARs,^14^ where replacing the N atom with a CH group at the C2 and C6 positions results in a drastic fall in hKMO inhibitory activity. Moreover, substituting with a COOH group at the fifth position enhances the potency of hKMOis (Figure 11). However, replacing this group with any other at the same position leads to a reduction in activity. Likewise, no substitution at the fourth position favors hKMO inhibitory activity.

**Figure 11:**
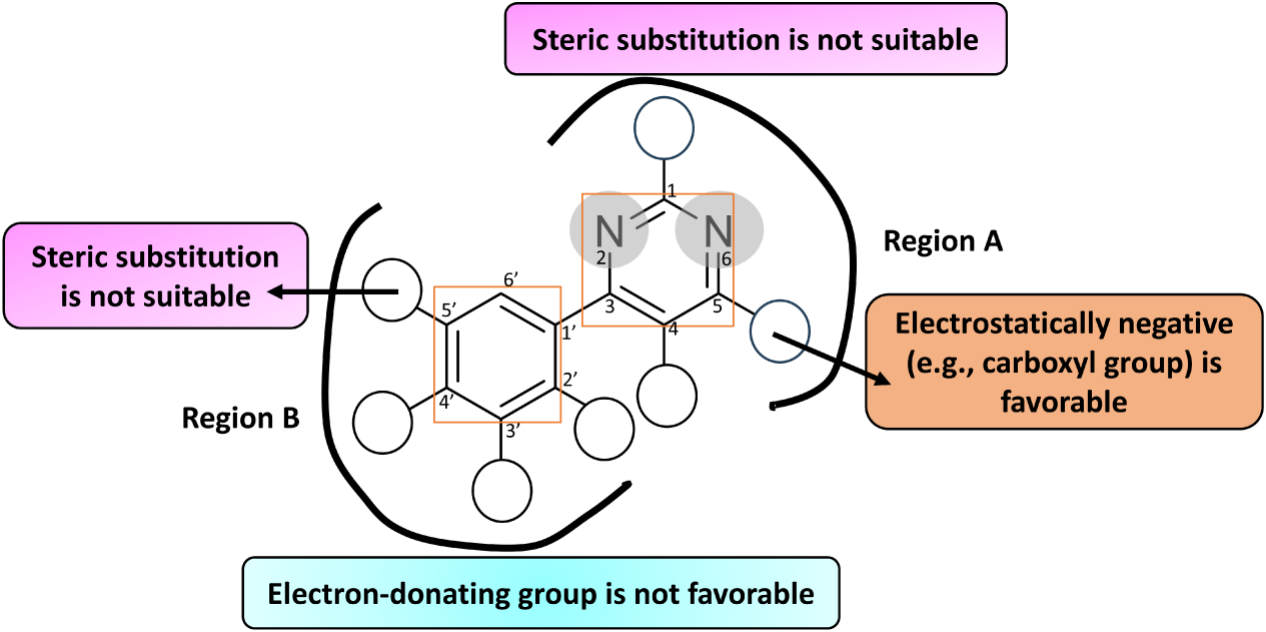
Key structural requirements of investigated derivatives for better hKMO inhibitory activity.

As suggested by the C and LJ descriptors, the introduction of a small group with negative charges may increase the activity near region B. Substitution at the C4’ position with any group other than a halogen may negatively impact hKMO inhibitory activity. Additionally, introducing a ring structure tends to decrease the effectiveness of hKMOis, indicating that nonhalogen substitutions and ring formations are generally unfavorable for maintaining or enhancing hKMO inhibitory activity levels in the molecule.

Methoxy substitution at the C3′ leads to a reduction in KMO inhibitory activity, indicating that while chlorine and fluoro groups support activity, the methoxy group diminishes the hKMO inhibitory potency. However, close to the C5′ substituent near region B, the introduction of steric groups may decrease the activity, and this observation is consistent with the experimental bioactivity. For instance, the substitution of a trifluoromethyl group at the C5′ position could be detrimental, potentially impairing the hKMO inhibitory activity (Figure 11).

## 4 Conclusion

This study provides valuable insights into the structural determinants of hKMO inhibitory activity through a combination of advanced computational techniques, including supervised ML, CSNs, and 3*D*/4D-QSAR studies. While it is true that the data set is relatively small, this limitation arises from the current lack of extensive publicly available data on experimentally validated hKMOis. Nevertheless, our study carefully addresses this by applying rigorous modeling techniques, cross-validation, and interpretable tools to maximize reliability and extract meaningful SARs from the available data. The developed supervised ML model demonstrates excellent performance, correctly classifying 89.29% of the instances in the validation data set. Further, the analysis of CSNs of hKMOis identified 14 distinct clusters, four of which contained more than 10 hKMOis. The remaining clusters are smaller, with two or fewer compounds per cluster.

For deeper insights, we focused on subgraph 1 (the cluster with the largest number of nodes) to develop the LQTA-QSAR model for the first time with the hKMOi data set, which provided valuable structural insights into the hKMOi space. The Coulomb (C) descriptors emphasize the importance of halogen atoms in enhancing hKMO inhibitory activity. This supports the hypothesis that halogen substitutions in key positions can lead to stronger binding interactions. The Lennard-Jones (LJ) descriptors, located near the pyrimidine nitrogen atom, provide critical structural information about the shape and flexibility of the ligands. Specifically, our analysis suggests that replacing the nitrogen atom with a methyl group (−CH_3_) at the C2 and C6 positions results in a significant reduction in the level of hKMO inhibition, aligning with previously observed SARs. In addition, the analysis of the C and LJ descriptors suggests several key trends for enhancing the hKMO inhibitory activity. For instance, introducing a small group with negative charges near region B may increase the activity. On the other hand, substitution at the C4’ position with anything other than a halogen atom appears to negatively impact hKMO inhibitory activity. Finally, the introduction of steric groups near region B (e.g., at the C5′ position) is found to decrease activity, consistent with experimental observations. Specifically, the substitution of a trifluoromethyl group at the C5′ position is identified as potentially detrimental, impairing the hKMO inhibitory activity. Apart from these details of SAR exploration, we have also identified important trends in the design and optimization of hKMOis by focusing on key molecular descriptors (ML and LQTA-QSAR) and their spatial relationships. For instance, the hKMO inhibitory potency can be enhanced by the incorporation of fluorinated benzene rings at Region B, likely due to their influence on molecular refractivity (as suggested by the descriptor *SMR_VSA7*). Hence, this fragment should be prioritized in the rational design of future hKMO inhibitors. In contrast, structural motifs such as multiple tertiary carbon atoms and mono- or dichloro-substituted benzene rings have been identified as negative contributors to hKMO inhibitory activity and therefore may be deprioritized in future hKMOi optimization strategies.

Additionaly, “**phKMOi_v1.0**” (available at https://phkmoiv1.streamlit.app/) is the first framework predicting KMO inhibitory activity that integrates an explainability support system (XAI). This tool also enhances the understanding of the role of specific molecular descriptors relating to KMO inhibitory activity, suggesting a potential link between the query molecule and its predictions.

## Supplementary Material



## Data Availability

The data associated with this study are contained within the article or in the Supporting Information. If required, the data will be made available on request. The Python scripts to calculate the Pearson correlation coefficients and other calculations have been provided at https://github.com/Amincheminform/phKMOi_v1.

## Notes

List of compounds with their molecule IDs, Smiles strings (Canonical Smiles), and hKMO inhibitory activities (IC_50_) (Table S1); list of hKMOis (with their molecule IDs) belongs to the subgraphs 1, 2, 4 and 12 (Table S2); pairs of connected nodes from subgraph 1, identified by their molecule IDs (Source_ID and Target_ID), along with the corresponding similarity values (*Tc*) between the nodes (Table S3); pairs of connected nodes from subgraph 2, identified by their molecule IDs (Source_ID and Target_ID), along with the corresponding similarity values (*Tc*) between the nodes (Table S4); pairs of connected nodes from subgraph 4, identified by their molecule IDs (Source_ID and Target_ID), along with the corresponding similarity values (*Tc*) between the nodes (Table S5); pairs of connected nodes from subgraph 12, identified by their molecule IDs (Source_ID and Target_ID), along with the corresponding similarity values (*Tc*) between the nodes (Table S6); results of 3D and 4D-QSAR models for hKMOis data set (Table S7); (A) Principal component analysis (PCA) of hKMOis: training (red) vs test sets (blue), (B) ROC plot of the test set hKMOis, and (C) feature importance plot of the selected descriptors as per the RF model (Figure S1); waterfall plots of the (i) query molecule, (ii) the most similar molecule from the data set with respect to the query molecule, and (iii) the most active molecule from the data set (Figure S2); a spring layout CSN component (*Tc* similarity variant) of 14 clusters (denoted as subgraphs) of hKMOis (Figure S3); a spring layout CSN component (*Tc* similarity variant) of all clusters of hKMOis together (Figure S4) (PDF)
