# Supplementary material for: First 4D-QSAR Study of Human Kynurenine 3 Monooxygenase (hKMO) Inhibitors: Integrating Chemical Space Networks and an Explainable Artificial Intelligence Platform for Neurodegenerative Disease Drug Discovery
Source: ACS Omega. 2025 Aug 29;10(35):39751–62. doi: 10.1021/acsomega.5c03404 (PMC12423829; doi:10.1021/acsomega.5c03404)
Supplement: Supplementary file 1 [file ao5c03404_si_002.pdf]

Supplementary File

First 4D-QSAR study of human kynurenine 3 monooxygenase (hKMO) inhibitors: Integrating chemical space networks and explainable artificial intelligence platform for neurodegenerative diseases drug discovery

Sk. Abdul Amin[1,*], Joao Pedro Gallo Almeida do Val[2], João Paulo Ataide Martins[2,*], Stefano Piotto[1]

[1]*Department of Pharmacy, Universita degli Studi di Salerno, Fisciano 84084 Campania, Italy*

[2]*Departamento de Química, Universidade Federal de Minas Gerais, 31270-901 Belo Horizonte-MG, Brazil*

*Corresponding authors:

Sk. Abdul Amin: pharmacist.amin@gmail.com

João Paulo Ataide Martins: joaopauloam@gmail.com

Table S1. List of compounds with their Molecule ID, Smiles strings (Canonical Smiles), and hKMO inhibitory activities (IC_{50}).

ID	Smiles	IC_{50} (nM)
K001	n1c(cc(nc1)C(=O)O)c1cccc(c1F)Cl	0.2
K002	n1c(cc(nc1)C(=O)O)c1ccc(c(c1)F)F	0.2
K003	n1c(cc(nc1)C(=O)O)c1ccc(c(c1)F)Cl	0.3
K004	n1c(cc(nc1)C(=O)O)c1cccc(c1)F	0.3
K005	n1c(cc(nc1)C(=O)O)c1cccc(c1)Cl	0.5
K006	n1c(cc(nc1)C(=O)O)c1ccc(c(c1)Cl)OC1CC1	0.5
K007	c1(c(ccc(c1)c1cc(ncn1)C(=O)O)Cl)OC1CC1	0.5
K008	n1c(cc(nc1)C(=O)O)c1ccc(c(c1)Cl)Cl	0.6
K009	n1c(cc(nc1)C(=O)O)c1ccc(c(c1)Cl)C	0.7
K010	n1c(cc(nc1)C(=O)O)c1ccc(c(c1)F)C	0.9
K011	n1c(cc(nc1)C(=O)O)c1ccc(c(c1)Cl)F	0.9
K012	n1c(cc(nc1)C(=O)O)c1ccc(c(c1)Cl)OC	1.2
K013	c1(c(cc2c(c1)oc(=O)n2CCC(=O)O)Cl)O[C@@H](c1nnccc1)C	1.3
K014	n1c(cc(nc1)C(=O)O)c1cc2c(cc1)occ2	1.5
K015	C(=O)(CCc1noc2c1cc(c(c2)O[C@H](C)c1ccc(cn1)C)Cl)O	1.6
K016	O=S(=O)(Nc1ccc(nn1)c1c(cccc1)N1CCCC1)c1cccc(c1)N(C)C	1.9
K017	C(=O)(CCn1c(=O)sc2c1cc(c(c2)O[C@H](C)c1ccccn1)Cl)O	2
K018	n1c(cc(nc1)c1n[nH]nn1)c1ccc(c(c1)Cl)Cl	2
K019	C(=O)(CCc1noc2c1cc(c(c2)O[C@H](C)c1ccc(nn1)C)Cl)O	2
K020	n1c(cc(nc1)C(=O)O)c1ccc(c(c1)Cl)OC1COC1	2
K021	O=S(=O)(Nc1ccc(nn1)c1cccc2c1N(CC2)C)c1ccc(cc1)C	2
K022	n1c(cc(nc1)C(=O)O)c1ccc(c(c1)Cl)OC(F)(F)F	2.1
K023	C(=O)(CCc1noc2c1cc(c(c2)O[C@H](C)c1ccccn1)Cl)O	2.3
K024	O=S(=O)(Nc1ccc(nn1)c1c(cccc1)N1CCCCC1)c1ccc(cc1)C	2.4
K025	C(=O)(CCn1c(=O)oc2c1cc(c(c2)O[C@H](C)c1ccc(cn1)F)Cl)O	2.5
K026	C(=O)(CCn1c(=O)oc2c1cc(c(c2)O[C@H](C)c1ccccn1)Cl)O	2.5
K027	C(=O)(CCc1noc2c1cc(c(c2)O[C@H](C)c1ccc(cn1)Cl)Cl)O	3.16
K028	C(=O)(CCc1noc2c1cc(c(c2)O[C@H](C)c1ccc(cn1)F)Cl)O	3.16
K029	C(=O)(CCn1c(=O)oc2c1cc(c(c2)O[C@H](C)c1ccc(cn1)Cl)Cl)O	3.2
K030	c1c(cc(nc1)C(=O)O)c1ccc(c(c1)Cl)Cl	3.2
K031	C(=O)(CCn1ncc2cc(c(cc12)Cl)O[C@H](C)c1ccccn1)O	3.2
K032	C(=O)(CCn1c(=O)oc2c1cc(c(c2)O[C@H](C)c1occn1)Cl)O	3.2
K033	C(=O)(CCn1c(=O)oc2c1cc(c(c2)O[C@H](C)c1ccc(cn1)C)Cl)O	3.2
K034	c1(c(cc2c(c1)oc(=O)n2CCC(=O)O)Cl)OC1CC1	3.2
K035	c1(c(cc2c(c1)oc(=O)n2CCC(=O)O)Cl)O[C@H](c1ncccc1)C	4
K036	O=S(=O)(Nc1ccc(nn1)c1c(cccc1)N1CCCC1)c1ccc(cc1)C	4.1
K037	O=S(=O)(Nc1ccc(c2c(ccc(c2)F)N2CCCCC2)nn1)c1ccc(cc1)C	4.2
K038	C(=O)(CCn1c(=O)oc2c1cc(c(c2)OCC)Cl)O	5
K039	C(=O)(CCn1ccc2cc(c(cc12)Cl)O[C@H](C)c1ccccn1)O	5
K040	C(=O)(CCn1c(=O)oc2c1cc(c(c2)O[C@H](C)c1cccc(n1)C)Cl)O	5
K041	n1c(cc(nc1)C(=O)O)c1ccc(c(c1)Cl)C(F)(F)F	5
K042	C(=O)(CCN1C(=O)COc2c1cc(c(c2)O[C@H](C)c1ccccn1)Cl)O	5

K043	C(=O)(CCn1c(=O)oc2c1cc(c(c2)O[C@H](C)c1ncccn1)Cl)O	5
K044	n1c(cc(nc1)C(=O)O)c1ccc(c(c1)Cl)OC(C)C	6
K045	n1c(c(c(nc1)C(=O)O)F)c1ccc(c(c1)Cl)Cl	6
K046	Clc1c(Cl)cc2nc(oc2c1)O	6
K047	O=S(=O)(Nc1ccc(c2c(cccc2F)N2CCCCC2)nn1)c1ccc(cc1)C	6.1
K048	C(=O)(CCn1c(=O)oc2c1cc(c(c2)Cl)Cl)O	6.3
K049	O=S(=O)(Nc1ccc(c2c(cc(cc2)F)N2CCCCC2)nn1)c1ccc(cc1)C	9.1
K050	c1(c(cc2c(c1)oc(=O)n2CCC(=O)O)Cl)CC	10
K051	n1c(c(c(nc1)C(=O)O)C)c1ccc(c(c1)Cl)Cl	10
K052	n1c(cc(nc1)C(=O)O)c1ccc(cc1)Cl	11
K053	n1c(cc(nc1C)C(=O)O)c1ccc(c(c1)Cl)Cl	12
K054	n1c(cc(nc1)C(=O)O)c1ccc(c(c1)Cl)OC1CCC1	12
K055	n1c(cc(nc1)c1[nH]c(=O)on1)c1ccc(c(c1)Cl)Cl	12
K056	O=S(=O)(Nc1ccc(c2c(cc(cc2)F)N2CCCCC2)nn1)CC1CCOCC1	13
K057	C(=O)(CCn1c(=O)oc2c1cc(c(c2)OC)Cl)O	13
K058	c1c(cc2c(c1)oc(=O)n2CCC(=O)O)Cl	13
K059	c1(c(cc2c(c1)oc(=O)[nH]2)Cl)C	13
K060	c1(c(cc2c(c1)oc(=O)n2CCC(=O)O)Cl)C	13
K061	C(=O)(CCn1c(=O)oc2c1cc(c(c2)CC(C)C)Cl)O	13
K062	O=S(=O)(Nc1ccc(c2c(cc(cc2)F)N2CCCCC2)nn1)C[C@H]1CCCO1	14
K063	n1c(cc(nc1)C(=O)O)c1cccc(c1)C	17
K064	O=S(=O)(Nc1ccc(c2c(cc(cc2)F)N2CCCCC2)nn1)C[C@H]1CCCOC1	18
K065	n1c(cc(nc1)C(=O)O)c1cc(cc(c1)Cl)Cl	19
K066	O=S(=O)(Nc1ccc(c2c(c(ccc2)F)N2CCCCC2)nn1)c1ccc(cc1)C	20
K067	c1c(c(cc(c1)C(=O)[C@H]1C[C@@H]1C(=O)O)Cl)Cl	20
K068	[C@@H]1([C@H](C1)C(=O)c1cc(c(cc1)Cl)Cl)C(=O)O	20
K069	c1c(cc2c(c1)onc2CCC(=O)O)Cl	25
K070	C(=O)(CCn1c(=O)oc2c1cc(c(c2)OCc1ccccc1)Cl)O	25
K071	c1(c(cc2c(c1)oc(=O)n2CCC(=O)O)Cl)OCC1CCC1	25
K072	n1c(cc(nc1)C(=O)NS(=O)(=O)c1ccccc1)c1ccc(c(c1)Cl)Cl	25
K073	O=S(=O)(Nc1ccc(c2c(cc(cc2)F)N2CCCCC2)nn1)CC(C)C	27
K074	O=S(=O)(Nc1ccc(c2c(cc(cc2)F)N2CCCCC2)nn1)CC1CCCC1	29
K075	n1c(c(c(nc1)C(=O)O)COC)c1ccc(c(c1)Cl)Cl	33
K076	c1cc(cc(c1)c1sc(nc1)NS(=O)(=O)c1ccc(c(c1)OC)OC)[N+](=O)[O-]	34
K077	O=S(=O)(Nc1ccc(c2c(cc(cc2)F)N2CCCCC2)nn1)C[C@@H]1CCCCO1	34
K078	O=S(=O)(Nc1ccc(c2c(cc(cc2)F)N2CCCCC2)nn1)CCC	37
K079	O=S(=O)(Nc1sc(c2cccc(c2)[N+](=O)[O-])cn1)c1ccc(cc1)C	37
K080	n1c(cc(nc1)C(=O)O)c1ccccc1	38
K081	n1c(cc(nc1)C(=O)O)c1ccc(cc1F)Cl	38
K082	O=S(=O)(Nc1ccc(c2c(cc(cc2)F)N2CCCCC2)nn1)CC(F)(F)F	39
K083	n1c(c(c(nc1)C(=O)O)OC)c1ccc(c(c1)Cl)Cl	40
K084	c1c(cc2c(c1)cnn2CCC(=O)O)Br	40
K085	n1c(cc(nc1)C(=O)O)c1ccc(c(c1)Cl)OCC1CC1	49
K086	n1c(cc(nc1)C(=O)O)c1ccc(c(c1)Cl)OC1CCCC1	57
K087	O=S(=O)(Nc1ccc(c2c(cc(cc2)F)N2CCCCC2)nn1)CCCOC	62
K088	c1c(cc2c(c1C)oc(=O)[nH]2)Cl	63

K089	n1c(cc(nc1)C(=O)O)c1ccc(cc1F)F	76
K090	c1(c(cc2c(c1)oc(=O)n2CCC(=O)O)Cl)OC(C)C	79
K091	c1c(cc2c(c1)OCC(=O)N2CCC(=O)O)Cl	79
K092	O(CCN1CCOCC1)c1ccc(c2ncnc(c2)C(=O)O)cc1Cl	83
K093	O=S(=O)(Nc1ccc(c2c(cc(cc2)F)N2CCCCC2)nn1)CCOC	84
K094	n1c(cc(cc1)C(=O)O)c1ccc(c(c1)Cl)Cl	90
K095	c1c(cc2c(c1)cnn2CC(=O)O)Cl	100
K096	c1(c(cc2c(c1)oc(=O)n2CCC(=O)O)Cl)C(C)C	100
K097	O=S(=O)(Nc1ccc(c2c(cc(cc2)F)N2CCCCC2)nn1)CC	116
K098	c1c(cc2c(c1)ccn2CCC(=O)O)Cl	126
K099	n1c(c(c(nc1)C(=O)O)O)c1ccc(c(c1)Cl)Cl	140
K100	C(=O)(c1cc(c(cc1)Cl)Cl)C[C@@H](C(=O)O)Cc1ccccc1	180
K101	C(=O)(C[C@@H](C(=O)O)N)c1cc(c(cc1)Cl)Cl	200
K102	n1c(c(c(nc1)C(=O)O)N)c1ccc(c(c1)Cl)Cl	260
K103	c1nc(cc(n1)NS(=O)(=O)c1ccccc1)c1ccc(c(c1)Cl)Cl	270
K104	N1(c2ccc(c3ncnc(c3)C(=O)O)cc2Cl)CCCC1	350
K105	n1c(cc(nc1)C(=O)O)c1ccc(c(c1)Cl)OC1CCCCC1	430
K106	c1c(cc2c(c1)nnn2CCC(=O)O)Cl	501
K107	c1c(cc2c(c1)oc(=O)n2CCC(=O)O)C#N	631
K108	c1c(cc2c(c1)cnn2C[C@@H](C(=O)O)O)Cl	631
K109	C(=O)(CCn1c(=O)oc2c1cc(c(c2)OCCN1CCCC1)Cl)O	794
K110	[N+](=O)(c1cc(C(=O)C[C@@H](C(=O)O)N)ccc1)[O-]	900
K111	n1c(cc(nc1)C(=O)O)c1cccnc1	990
K112	c1c(cc2c(c1)oc(=O)n2CCC(=O)O)OC	1,000
K113	c1(ccc2c(c1)cnn2CCC(=O)O)Cl	1,000
K114	C(=O)(CCn1c(=O)oc2c1cc(c(c2)OCC1CC1)Cl)O	1,580
K115	n1c(cc(nc1)N)c1ccc(c(c1)Cl)Cl	1,700
K116	c1c(cc2c(c1)c(nn2CCC(=O)O)C)Cl	1,995
K117	n1c(cc(nc1)c1nnnn1C)c1ccc(c(c1)Cl)Cl	2,300
K118	c1c(cc2c(c1)cnn2C[C@@H](C(=O)O)N)Cl	2,512
K119	c1c(cc2c(c1)cnn2C[C@@H](C(=O)O)C)Cl	2,512
K120	c1(c(cc2c(c1C)N(C(=O)CO2)CC(=O)O)C)Cl	2,600
K121	c1c(cc2c(c1)cnn2CCCC(=O)O)Cl	3,162
K122	c1c(cc2c(c1)cnn2CCC(=O)O)C	3,162
K123	C(=O)(C[C@@H](C(=O)O)O)c1cc(c(cc1)Cl)Cl	3,300
K124	n1c(cc(nc1)C#N)c1ccc(c(c1)Cl)Cl	4,000
K125	C(=O)(CCn1c(=O)oc2c1cc(c(c2)OCc1ccccn1)Cl)O	5,010
K126	c1c(cc2c(c1)cnn2[C@H](CC(=O)O)C)Cl	5,012
K127	c1c(cc2c(c1)oc(=O)n2CCC(=O)O)C(F)(F)F	5,012
K128	n1c(cc(nc1)c1ncon1)c1ccc(c(c1)Cl)Cl	5,060
K129	c1ccc2c(c1)n(nc2)CCC(=O)O	6,310
K130	c1ccc(cc1[N+](=O)[O-])c1c(sc(n1)NS(=O)(=O)c1ccc(c(c1)OC)OC)CN1CCCCC1	11,000
K131	c1(c(cc2c(c1)oc(=O)n2CCC(=O)O)Cl)C(F)(F)F	15,849
K132	c1ccc(cc1[N+](=O)[O-])c1c(sc(n1)NS(=O)(=O)c1ccc(c(c1)OC)OC)CN	17,000
K133	C(=O)(CCn1c(=O)oc2c1cc(c(c2)O[C@H](C)c1cc(ccn1)C)Cl)O	31,623
K134	c1ccc(cc1[N+](=O)[O-])c1c(sc(n1)NS(=O)(=O)c1ccc(c(c1)OC)OC)CO	39,000

K135	C(=O)(CCn1c(=O)oc2c1cc(c(c2)OCCOC)Cl)O	50,119
K136	C(=O)(CCn1c(=O)oc2c1cc(c(c2)O[C@@H](C)c1ccccn1)Cl)O	63,096
K137	c1(c(cc2c(c1)oc(=O)n2CCC(=O)O)Cl)C#N	79,433

Table S2. List of hKMOis (with their Molecule ID) belongs to the subgraph 1, 2, 4 and 12.

Molecule ID	Subgraph
K001	1
K002	1
K003	1
K004	1
K005	1
K006	1
K007	1
K008	1
K009	1
K010	1
K011	1
K012	1
K014	1
K020	1
K022	1
K041	1
K044	1
K045	1
K051	1
K052	1
K053	1
K054	1
K063	1
K065	1
K075	1
K080	1
K081	1
K083	1
K085	1
K086	1
K089	1
K092	1
K099	1
K102	1
K104	1
K105	1
K111	1
K124	1
K040	2
K025	2
K125	2
K048	2
K071	2

K136	2
K050	2
K013	2
K038	2
K060	2
K026	2
K090	2
K135	2
K070	2
K112	2
K131	2
K033	2
K035	2
K114	2
K096	2
K127	2
K043	2
K133	2
K032	2
K029	2
K109	2
K107	2
K061	2
K137	2
K058	2
K034	2
K057	2
K077	4
K021	4
K024	4
K097	4
K016	4
K093	4
K062	4
K049	4
K073	4
K056	4
K078	4
K066	4
K047	4
K082	4
K074	4
K036	4
K037	4
K087	4
K064	4

K095	12
K113	12
K122	12
K108	12
K116	12
K084	12
K119	12
K129	12
K118	12
K121	12
K126	12

Table S3. Pairs of connected nodes from subgraph 1, identified by their Molecule IDs (Source_ID and Target_ID), along with the corresponding similarity values (*Tc*) between the nodes.

Source_ID	Target_ID	*Tc*
K105	K086	0.983
K054	K086	0.974
K005	K065	0.971
K006	K044	0.97
K054	K105	0.963
K054	K006	0.959
K054	K044	0.952
K054	K085	0.945
K006	K086	0.945
K044	K020	0.944
K012	K044	0.943
K008	K005	0.94
K006	K085	0.94
K086	K044	0.937
K105	K006	0.934
K022	K012	0.931
K086	K085	0.931
K002	K004	0.93
K085	K044	0.93
K008	K065	0.927
K105	K044	0.927
K006	K020	0.922
K105	K085	0.92
K006	K012	0.915
K054	K020	0.911
K080	K052	0.908
K005	K009	0.903
K085	K012	0.903
K004	K010	0.9
K054	K012	0.898
K086	K020	0.898
K105	K020	0.891
K012	K020	0.89
K092	K044	0.89
K085	K020	0.888
K041	K009	0.885
K022	K044	0.884
K086	K012	0.884
K099	K083	0.884

K005	K052	0.883
K008	K052	0.88
K065	K009	0.88
K005	K011	0.877
K003	K004	0.877
K063	K080	0.876
K105	K012	0.874
K051	K075	0.871
K006	K092	0.868
K012	K092	0.864
K008	K009	0.86
K006	K022	0.859
K065	K052	0.858
K085	K092	0.858
K092	K020	0.858
K008	K053	0.855
K054	K092	0.855
K001	K081	0.854
K011	K065	0.854
K022	K085	0.851
K005	K080	0.85
K080	K004	0.85
K054	K022	0.849
K002	K010	0.846
K086	K092	0.846
K005	K012	0.842
K022	K020	0.842
K089	K081	0.842
K105	K092	0.837
K022	K086	0.837
K008	K011	0.833
K105	K022	0.831
K065	K080	0.825
K002	K003	0.825
K065	K012	0.824
K001	K011	0.822
K003	K052	0.821
K022	K092	0.819
K003	K010	0.815
K063	K052	0.811
K111	K080	0.808
K011	K009	0.807
K001	K089	0.806
K052	K009	0.806

K008	K012	0.805
K005	K053	0.804
K011	K003	0.801
K008	K080	0.799
K005	K041	0.799
K006	K007	0.799
K065	K053	0.796
K005	K044	0.794
K080	K002	0.791
K002	K089	0.789
K004	K052	0.789
K008	K102	0.785
K005	K022	0.784
K008	K051	0.783
K008	K099	0.781
K065	K044	0.781
K065	K041	0.78
K011	K052	0.777
K012	K009	0.775
K005	K063	0.774
K003	K081	0.774
K007	K044	0.772
K001	K005	0.771
K008	K041	0.77
K005	K006	0.77
K008	K045	0.768
K054	K007	0.768
K065	K022	0.768
K080	K009	0.768
K008	K044	0.766
K089	K004	0.766
K063	K004	0.765
K080	K010	0.765
K011	K012	0.763
K052	K081	0.762
K005	K085	0.76
K006	K065	0.758
K086	K007	0.758
K054	K005	0.756
K053	K009	0.755
K001	K065	0.754
K065	K063	0.753
K052	K053	0.753
K085	K007	0.752

K008	K022	0.751
K065	K085	0.751
K005	K020	0.75
K105	K007	0.75
K008	K003	0.749
K008	K006	0.749
K063	K009	0.749
K111	K052	0.747
K011	K002	0.747
K011	K004	0.747
K052	K012	0.747
K005	K004	0.745
K011	K080	0.745
K080	K003	0.745
K007	K020	0.745
K054	K065	0.744
K005	K086	0.744
K063	K010	0.744
K008	K001	0.742
K005	K003	0.742
K065	K020	0.74
K008	K085	0.739
K005	K102	0.738
K002	K052	0.737
K005	K051	0.736
K005	K105	0.736
K065	K086	0.735
K044	K009	0.735
K005	K099	0.734
K008	K054	0.733
K008	K063	0.732
K011	K041	0.732
K011	K053	0.732
K051	K065	0.732
K065	K099	0.732
K065	K102	0.732
K011	K044	0.731
K080	K089	0.731
K011	K081	0.73
K022	K009	0.73
K008	K020	0.729
K011	K022	0.729
K007	K012	0.728
K001	K003	0.727

K005	K092	0.727
K105	K065	0.727
K065	K004	0.726
K052	K010	0.726
K111	K063	0.725
K001	K009	0.724
K065	K003	0.724
K008	K086	0.722
K005	K045	0.722
K011	K089	0.722
K008	K081	0.721
K012	K053	0.721
K014	K080	0.718
K065	K045	0.718
K063	K002	0.717
K041	K052	0.717
K006	K009	0.716
K065	K092	0.716
K080	K012	0.716
K111	K004	0.715
K041	K012	0.715
K008	K105	0.714
K005	K104	0.714
K053	K102	0.714
K008	K004	0.713
K011	K006	0.713
K111	K005	0.712
K005	K081	0.712
K089	K010	0.712
K008	K124	0.711
K004	K081	0.709
K008	K092	0.708
K065	K104	0.708
K003	K089	0.708
K099	K053	0.708
K085	K009	0.708
K011	K085	0.707
K054	K009	0.706
K052	K044	0.706
K054	K011	0.705
K007	K092	0.705
K053	K045	0.704
K005	K002	0.702
K051	K053	0.702

K010	K009	0.701
K009	K020	0.701
K011	K010	0.7
K007	K052	0.7
K051	K009	0.698
K002	K081	0.698
K011	K086	0.697
K022	K007	0.697
K102	K009	0.697
K065	K081	0.696
K022	K052	0.696
K086	K009	0.696
K099	K009	0.696
K011	K020	0.695
K111	K065	0.694
K041	K053	0.694
K044	K053	0.693
K080	K081	0.692
K009	K045	0.692
K008	K083	0.691
K001	K012	0.691
K011	K045	0.691
K011	K099	0.691
K052	K102	0.691
K008	K104	0.69
K011	K051	0.69
K011	K105	0.69
K022	K041	0.69
K003	K009	0.69
K089	K052	0.69
K011	K063	0.689
K105	K009	0.689
K051	K052	0.689
K001	K052	0.688
K005	K010	0.687
K006	K052	0.687
K041	K044	0.687
K099	K052	0.687
K014	K063	0.686
K065	K002	0.685
K099	K012	0.684
K080	K053	0.683
K008	K075	0.682
K014	K052	0.682

K063	K003	0.682
K011	K102	0.681
K006	K053	0.681
K022	K053	0.681
K004	K009	0.681
K012	K102	0.681

Tanimoto coefficient (*Tc*) calculated from RDKit topological fingerprints.

Table S4. Pairs of connected nodes from subgraph 2, identified by their Molecule IDs (Source_ID and Target_ID), along with the corresponding similarity values (*Tc*) between the nodes.

Source_ID	Target_ID	*Tc*
K026	K035	1
K026	K136	1
K035	K136	1
K038	K090	0.989
K090	K034	0.984
K114	K071	0.98
K038	K135	0.979
K026	K040	0.978
K035	K040	0.978
K136	K040	0.978
K038	K034	0.974
K038	K057	0.974
K096	K050	0.973
K029	K026	0.969
K029	K035	0.969
K029	K136	0.969
K061	K050	0.969
K090	K135	0.969
K038	K114	0.967
K026	K133	0.966
K035	K133	0.966
K133	K136	0.966
K026	K025	0.965
K026	K033	0.965
K033	K035	0.965
K033	K136	0.965
K035	K025	0.965
K136	K025	0.965
K057	K090	0.964
K114	K034	0.962
K038	K071	0.959
K090	K114	0.959
K060	K050	0.956
K135	K034	0.956
K057	K135	0.954
K029	K040	0.951
K090	K071	0.951
K071	K034	0.951
K137	K060	0.949
K033	K040	0.949
K057	K034	0.949

K135	K114	0.949
K133	K040	0.947
K025	K040	0.947
K096	K061	0.944
K033	K133	0.943
K057	K114	0.942
K135	K071	0.942
K029	K033	0.939
K029	K025	0.938
K029	K133	0.937
K038	K109	0.937
K133	K025	0.935
K033	K025	0.934
K057	K071	0.934
K060	K096	0.931
K090	K109	0.93
K060	K061	0.927
K026	K125	0.925
K035	K125	0.925
K125	K136	0.925
K070	K057	0.923
K135	K109	0.92
K034	K109	0.918
K070	K125	0.914
K057	K109	0.913
K114	K109	0.913
K137	K050	0.909
K071	K109	0.908
K060	K131	0.907
K125	K040	0.904
K038	K070	0.901
K048	K058	0.9
K029	K125	0.896
K026	K013	0.895
K035	K013	0.895
K013	K136	0.895
K125	K025	0.893
K125	K133	0.893
K070	K090	0.892
K033	K125	0.892
K070	K135	0.887
K137	K096	0.886
K032	K043	0.886
K137	K061	0.885
K026	K043	0.885
K057	K125	0.885

K035	K043	0.885
K043	K136	0.885
K070	K034	0.881
K013	K040	0.881
K038	K043	0.88
K131	K050	0.878
K070	K114	0.875
K107	K127	0.873
K043	K090	0.873
K038	K125	0.872
K070	K071	0.872
K033	K013	0.872
K133	K013	0.871
K029	K013	0.87
K013	K025	0.869
K137	K131	0.868
K043	K040	0.868
K060	K058	0.864
K125	K090	0.863
K043	K034	0.863
K043	K135	0.863
K029	K043	0.86
K061	K131	0.859
K125	K135	0.859
K096	K131	0.858
K033	K043	0.858
K133	K043	0.858
K043	K025	0.858
K057	K043	0.857
K043	K114	0.857
K125	K043	0.856
K125	K034	0.855
K043	K071	0.855
K125	K013	0.853
K070	K109	0.852
K026	K070	0.851
K070	K035	0.851
K070	K136	0.851
K026	K032	0.85
K032	K035	0.85
K032	K136	0.85
K125	K114	0.848
K038	K032	0.847
K070	K013	0.847
K070	K043	0.847
K043	K013	0.847

K125	K071	0.846
K032	K090	0.843
K038	K026	0.839
K038	K035	0.839
K038	K136	0.839
K032	K040	0.839
K043	K109	0.839
K038	K013	0.836
K070	K040	0.836
K032	K034	0.835
K032	K135	0.835
K090	K013	0.834
K026	K090	0.832
K035	K090	0.832
K125	K109	0.832
K090	K136	0.832
K032	K114	0.831
K029	K032	0.83
K032	K025	0.83
K026	K135	0.828
K070	K033	0.828
K070	K133	0.828
K035	K135	0.828
K135	K136	0.828
K029	K070	0.827
K057	K058	0.827
K032	K033	0.826
K032	K125	0.826
K032	K133	0.826
K013	K034	0.826
K058	K050	0.826
K032	K057	0.825
K032	K071	0.825
K135	K013	0.825
K026	K034	0.824
K035	K034	0.824
K136	K034	0.824
K032	K013	0.823
K032	K109	0.822
K070	K025	0.822
K038	K040	0.821
K137	K058	0.82
K026	K071	0.82
K035	K071	0.82
K136	K071	0.82
K026	K114	0.819

K035	K114	0.819
K136	K114	0.819
K026	K057	0.818
K032	K070	0.818
K057	K035	0.818
K057	K136	0.818
K013	K071	0.817
K013	K114	0.816
K057	K013	0.815
K090	K040	0.814
K029	K038	0.813
K038	K133	0.811
K038	K025	0.81
K038	K033	0.81
K026	K109	0.81
K035	K109	0.81
K135	K040	0.81
K136	K109	0.81
K040	K034	0.807
K029	K090	0.806
K038	K058	0.806
K090	K025	0.805
K029	K135	0.804
K096	K058	0.804
K133	K090	0.804
K013	K109	0.804
K033	K090	0.803
K133	K135	0.802
K040	K071	0.802
K040	K114	0.802
K033	K135	0.801
K135	K025	0.801
K061	K058	0.8
K057	K040	0.8
K029	K034	0.799
K029	K071	0.798
K133	K034	0.798
K025	K034	0.798
K060	K048	0.797
K033	K034	0.797
K090	K058	0.797
K029	K114	0.796
K033	K071	0.796
K133	K071	0.796
K133	K114	0.795
K033	K114	0.794

K040	K109	0.793
K029	K057	0.792
K025	K071	0.792
K025	K114	0.792
K029	K109	0.79
K057	K025	0.79
K057	K133	0.79
K033	K057	0.789
K135	K058	0.789
K025	K109	0.788
K133	K109	0.787
K058	K034	0.785
K131	K058	0.783
K033	K109	0.782
K114	K058	0.779
K057	K048	0.773
K071	K058	0.773
K048	K050	0.769
K070	K058	0.763
K137	K048	0.76
K038	K048	0.758
K060	K057	0.757
K058	K109	0.755
K061	K048	0.754
K096	K048	0.753
K090	K048	0.752
K135	K048	0.747
K048	K034	0.745
K038	K060	0.744
K060	K070	0.741
K114	K048	0.74
K060	K090	0.739
K112	K058	0.739
K131	K048	0.738
K057	K050	0.736
K137	K057	0.735
K060	K135	0.735
K048	K071	0.734
K060	K034	0.732
K107	K058	0.731
K125	K058	0.731
K070	K048	0.729
K070	K050	0.728
K038	K050	0.726
K137	K070	0.725
K060	K114	0.725

K096	K057	0.724
K038	K137	0.723
K060	K071	0.722
K061	K057	0.721
K090	K050	0.721
K107	K112	0.72
K137	K090	0.72
K048	K109	0.72
K135	K050	0.719
K096	K070	0.718
K131	K057	0.717
K137	K135	0.716
K061	K070	0.716
K034	K050	0.716
K038	K096	0.715
K038	K061	0.714
K038	K131	0.714
K137	K034	0.713
K114	K050	0.712
K060	K125	0.711
K096	K090	0.71
K058	K127	0.71
K096	K135	0.709
K061	K090	0.709
K131	K090	0.709
K043	K058	0.709
K061	K135	0.708
K112	K057	0.708
K071	K050	0.708
K137	K114	0.707
K061	K034	0.707
K070	K131	0.707
K131	K135	0.707
K060	K109	0.706
K096	K034	0.705
K137	K071	0.704
K131	K034	0.704
K125	K048	0.704
K061	K114	0.702
K096	K114	0.7
K131	K114	0.7
K038	K112	0.699
K061	K071	0.699
K131	K071	0.699
K125	K050	0.699
K096	K071	0.697

K112	K048	0.696
K131	K127	0.696
K137	K125	0.695
K112	K127	0.695
K112	K090	0.694
K107	K137	0.693
K107	K060	0.692
K137	K109	0.692
K109	K050	0.692
K043	K048	0.691
K096	K125	0.69
K061	K125	0.689
K131	K109	0.688
K060	K127	0.686
K112	K034	0.686
K112	K135	0.686
K061	K109	0.685
K112	K114	0.685
K107	K048	0.684
K131	K125	0.684
K032	K058	0.682
K096	K109	0.681
K112	K071	0.681

Tanimoto coefficient (*Tc*) calculated from RDKit topological fingerprints.

Table S5. Pairs of connected nodes from subgraph 4, identified by their Molecule IDs (Source_ID and Target_ID), along with the corresponding similarity values (*Tc*) between the nodes.

Source_ID	Target_ID	*Tc*
K078	K073	0.986
K036	K024	0.986
K078	K097	0.985
K078	K087	0.978
K077	K062	0.974
K073	K097	0.971
K097	K093	0.968
K087	K073	0.966
K097	K082	0.965
K087	K097	0.964
K074	K073	0.958
K078	K093	0.954
K078	K082	0.953
K074	K056	0.945
K074	K078	0.944
K087	K093	0.944
K073	K093	0.943
K073	K082	0.942
K073	K056	0.94
K082	K093	0.937
K087	K082	0.935
K049	K024	0.933
K074	K097	0.93
K037	K024	0.93
K037	K066	0.928
K074	K087	0.926
K078	K056	0.926
K056	K064	0.925
K087	K056	0.923
K074	K064	0.921
K036	K049	0.92
K036	K037	0.917
K049	K037	0.917
K097	K056	0.913
K087	K064	0.911
K049	K047	0.908
K074	K082	0.907
K074	K093	0.904
K073	K064	0.904
K093	K062	0.904
K036	K016	0.9

K093	K056	0.897
K077	K093	0.894
K082	K056	0.893
K078	K064	0.891
K037	K047	0.89
K078	K062	0.888
K016	K024	0.888
K049	K066	0.887
K087	K062	0.886
K066	K024	0.885
K073	K062	0.881
K074	K062	0.879
K078	K077	0.879
K097	K064	0.878
K087	K077	0.876
K097	K062	0.875
K074	K077	0.874
K047	K024	0.874
K064	K062	0.874
K036	K066	0.873
K047	K066	0.872
K077	K056	0.871
K077	K073	0.871
K056	K062	0.871
K093	K064	0.868
K077	K064	0.867
K077	K097	0.866
K082	K064	0.865
K036	K047	0.862
K082	K062	0.857
K077	K082	0.848
K049	K016	0.841
K037	K016	0.837
K036	K021	0.804
K021	K024	0.801
K047	K016	0.799
K016	K066	0.799
K049	K097	0.769
K049	K021	0.767
K037	K021	0.765
K049	K087	0.762
K078	K049	0.761
K049	K073	0.757
K049	K093	0.752
K049	K082	0.751
K021	K016	0.751

K074	K049	0.744
K021	K066	0.741
K049	K056	0.74
K021	K047	0.734
K049	K064	0.733
K049	K062	0.722
K049	K077	0.716
K087	K047	0.712
K097	K047	0.708
K073	K047	0.707
K097	K037	0.707
K078	K047	0.706
K097	K024	0.706
K087	K037	0.703
K078	K037	0.702
K087	K024	0.7
K073	K037	0.7
K078	K024	0.699
K047	K064	0.699
K036	K097	0.698
K047	K082	0.698
K047	K056	0.697
K074	K047	0.696
K073	K024	0.696
K047	K093	0.696
K036	K087	0.693
K097	K016	0.693
K037	K082	0.693
K037	K093	0.692
K078	K036	0.691
K074	K037	0.69
K024	K082	0.69
K024	K093	0.69
K087	K066	0.689
K097	K066	0.689
K036	K073	0.688
K078	K066	0.687
K087	K016	0.687
K073	K066	0.687
K037	K056	0.687
K078	K016	0.686
K047	K062	0.686
K074	K024	0.684
K073	K016	0.684
K016	K093	0.684
K036	K093	0.683

K036	K082	0.682
K037	K064	0.682
K024	K056	0.681
K077	K047	0.68
K066	K082	0.68

Tanimoto coefficient (*Tc*) calculated from RDKit topological fingerprints.

Table S6. Pairs of connected nodes from subgraph 12, identified by their Molecule IDs (Source_ID and Target_ID), along with the corresponding similarity values (*Tc*) between the nodes.

**Source_ID**	**Target_ID**	***Tc***
K113	K129	0.858
K129	K084	0.829
K129	K122	0.826
K118	K119	0.819
K119	K126	0.816
K119	K108	0.815
K119	K121	0.797
K118	K108	0.784
K126	K108	0.781
K121	K126	0.776
K118	K126	0.774
K118	K121	0.759
K113	K119	0.751
K119	K129	0.75
K121	K108	0.75
K113	K084	0.744
K095	K108	0.741
K116	K119	0.739
K113	K122	0.736
K095	K121	0.736
K113	K118	0.724
K084	K122	0.714
K116	K118	0.713
K116	K108	0.711
K116	K126	0.711
K113	K108	0.71
K113	K126	0.71
K118	K129	0.709
K129	K126	0.709
K129	K108	0.706
K119	K095	0.704
K113	K121	0.688
K121	K129	0.685
K116	K121	0.682

Tanimoto coefficient (*Tc*) calculated from RDKit topological fingerprints.

Table S7. Results of 3D and 4D-QSAR models for hKMOis dataset.

Parameters	3D-QSAR	4D-QSAR
R^2	0.8697	0.9196
$PRESS_{CV}$	8.7081	5.6356
Q^2	0.8119	0.8661
F	42.7071	154.3708
R^2_{Pred} (Q^2F_1)	0.8073	0.5875
Q^2F_2	0.7609	0.5872
MAE	0.3465	0.4107
nLV	05	02

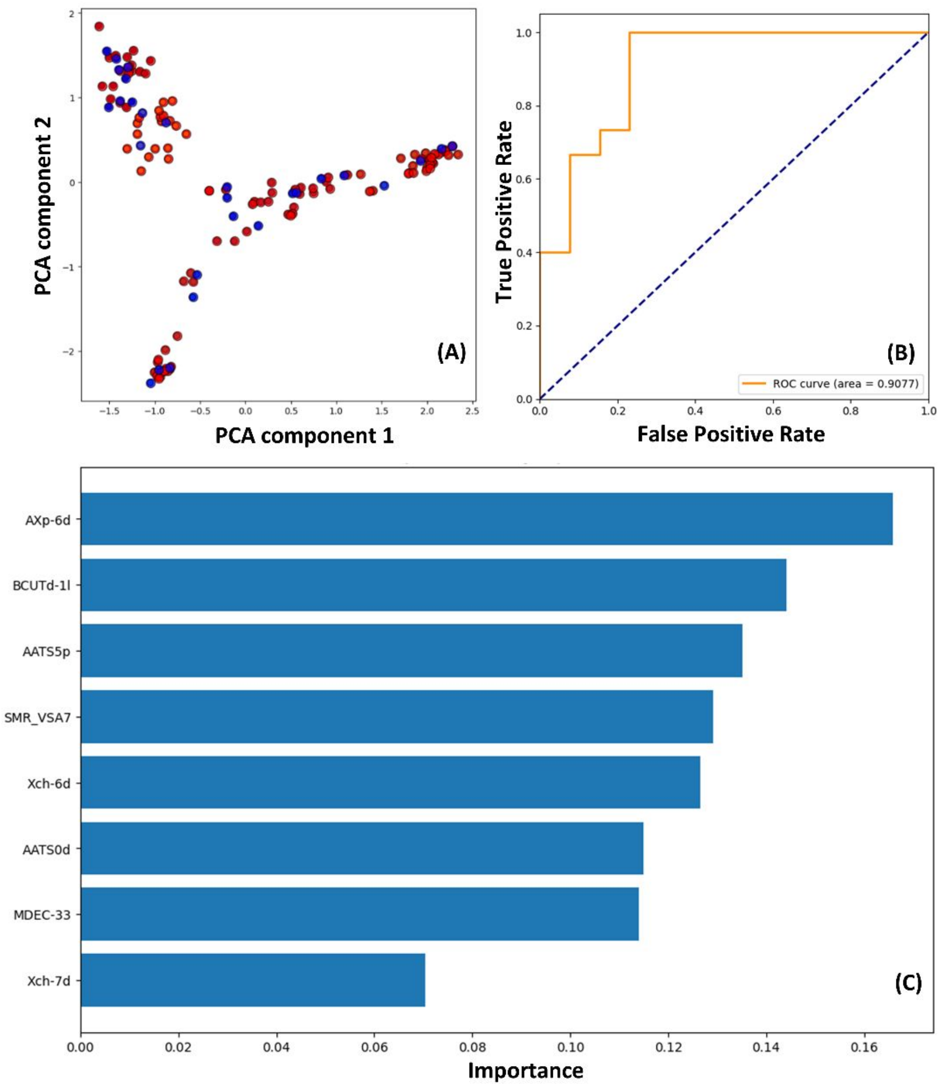

Figure S1. **(A)** Principal component analysis (PCA) of hKMOis: training (red) vs test sets (blue), **(B)** ROC plot of the test set hKMOis, and **(C)** Feature importance plot of the selected descriptors as per the RF model.

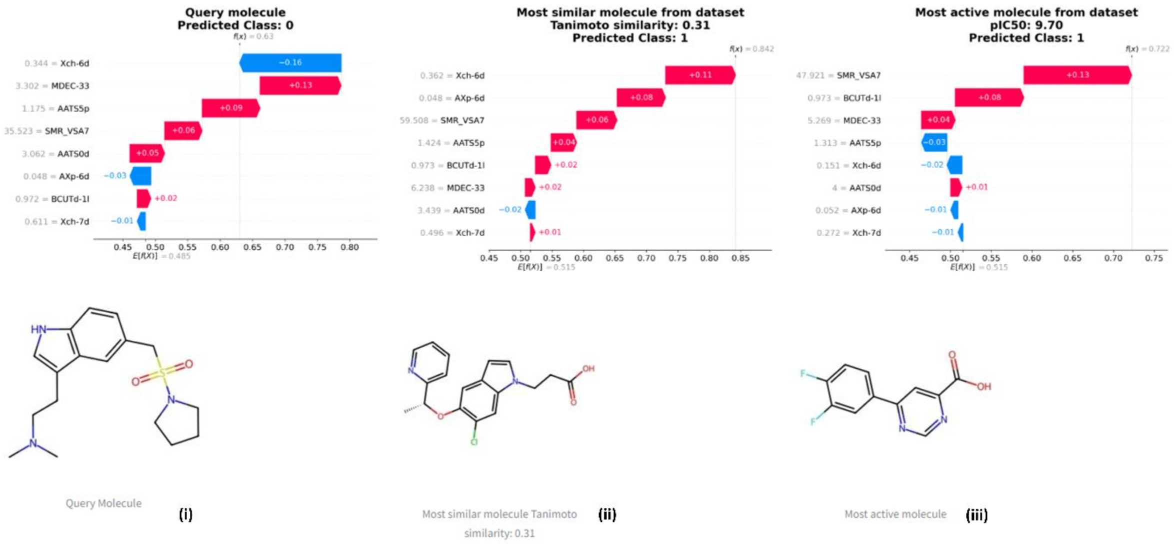

Figure S2. Water falls plots of the (i) query molecule, (ii) the most similar molecule from the dataset with respect to the query molecule, (iii) the most active molecule from the dataset.

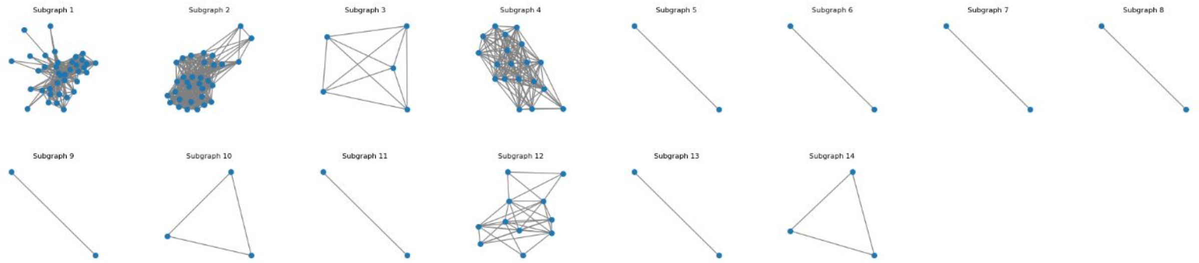

Figure S3. A spring layout CSNs component (*Tc* Similarity variant) of 14 clusters (denoted as Subgraphs) of hKMOis.

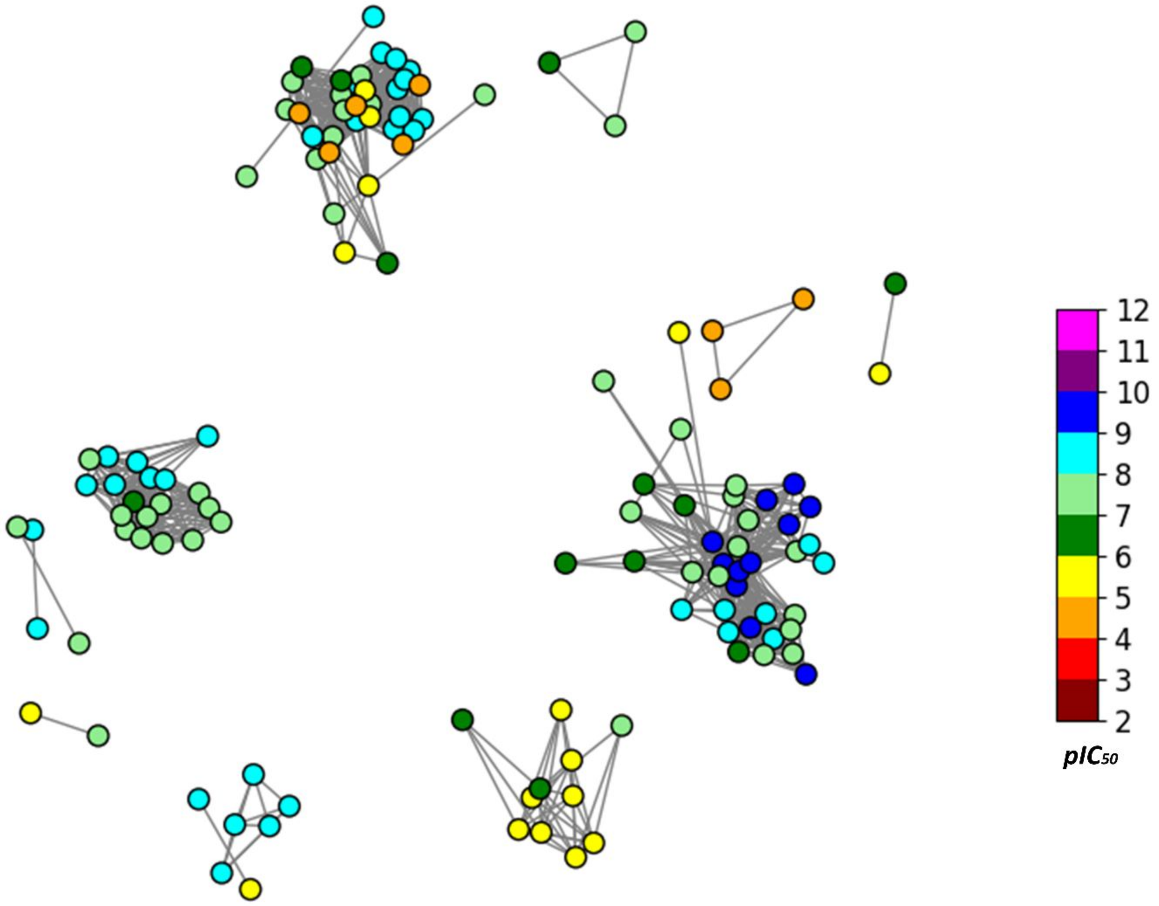

Figure S4. A spring layout CSNs component (*Tc* Similarity variant) of all clusters of hKMOis together.
